# Machine learning reveals the dynamic importance of accessory sequences for *Salmonella* outbreak clustering

**DOI:** 10.1128/mbio.02650-24

**Published:** 2025-01-28

**Authors:** Chao Chun Liu, William W. L. Hsiao

**Affiliations:** 1 Department of Molecular Biology and Biochemistry, Simon Fraser University, Burnaby, British Columbia, Canada; 2 Faculty of Health Sciences, Simon Fraser University, Burnaby, British Columbia, Canada; University of Georgia Center for Food Safety, Griffin, Georgia, USA; University of Maryland, College Park, Maryland, USA

**Keywords:** *Salmonella*, microbial genomics, molecular microevolution, outbreak clustering, machine learning

## Abstract

Bacterial typing at whole-genome scales is now feasible owing to decreasing costs in high-throughput sequencing and the recent advances in computation. The unprecedented resolution of whole-genome typing is achieved by genotyping the variable segments of bacterial genomes that can fluctuate significantly in gene content. However, due to the transient and hypervariable nature of many accessory elements, the value of the added resolution in outbreak investigations remains disputed. To assess the analytical value of bacterial accessory genomes in clustering epidemiologically related cases, we trained classifiers on a set of genomes collected from 24 *Salmonella enterica* outbreaks of food, animal, or environmental origin. The models demonstrated high precision and recall on unseen test data with near-perfect accuracy in classifying clonal and short-term outbreaks. Annotating the genomic features important for cluster classification revealed functional enrichment of molecular fingerprints in genes involved in membrane transportation, trafficking, and carbohydrate metabolism. Importantly, we discovered polymorphisms in mobile genetic elements (MGEs) and gain/loss of MGEs to be informative in defining outbreak clusters. To quantify the ability of MGE variations to cluster outbreak clones, we devised a reference-free tree-building algorithm inspired by colored de Bruijn graphs, which enabled topological comparisons between MGE and standard typing methods. Systematic evaluation of clustering MGEs on an unseen dataset of 34 *Salmonella* outbreaks yielded mixed results that exemplified the power of accessory sequence variations when core genomes of unrelated cases are insufficiently discriminatory, as well as the distortion of outbreak signals by microevolution events or the incomplete assembly of MGEs.

**IMPORTANCE:**

Gene-by-gene typing is widely used to detect clusters of foodborne illnesses that share a common origin. It remains actively debated whether the inclusion of accessory sequences in bacterial typing schema is informative or deleterious for cluster definitions in outbreak investigations due to the potential confounding effects of horizontal gene transfer. By training machine learning models on a curated set of historical *Salmonella* outbreaks, we revealed an enriched presence of outbreak distinguishing features in a wide range of mobile genetic elements. Systematic comparison of the efficacy of clustering different accessory elements against standard sequence typing methods led to our cataloging of scenarios where accessory sequence variations were beneficial and uninformative to resolving outbreak clusters. The presented work underscores the complexity of the molecular trends in enteric outbreaks and seeks to inspire novel computational ways to exploit whole-genome sequencing data in enteric disease surveillance and management.

## INTRODUCTION

Timely and targeted intervention of foodborne pathogen transmission requires active monitoring of the incidences and demographics of illnesses and environmental contamination. Detecting acute surges in suspected foodborne illnesses provides early warning signals of active transmissions among one or more populations (1). As foodborne outbreaks are frequently associated with the accelerated propagation of a single clone, manifesting clusters of characteristically similar cases, evidence of clonality is widely used to link reported cases and detect outbreak clusters (2). Cluster detection operates on the observation that individual strains are molecularly distinct, thereby enabling the discrimination of related and unrelated cases by analyzing molecular differences (2). Since the inception of molecular subtyping, methods to infer clonality have progressively improved from detecting conspicuous patterns produced by restriction enzymes, i.e., pulsed-field gel electrophoresis (PFGE), and cell lysis, i.e., phage typing, to interrogating nucleotide-level changes enabled by whole-genome sequencing (WGS). The capacity to discriminate strains differing by as little as one nucleotide in conjunction with decreasing sequencing costs has triggered the widespread adoption of WGS by laboratory-based surveillance networks (e.g., PulseNet) and public health agencies (e.g., UK Health Security Agency and Public Health Agency of Canada) to rapidly identify high-resolution clusters that were cryptic to previously deployed methods (3–5).

However, with the promising outlook of applying genomics to improve foodborne pathogen surveillance and outbreak investigations, came new challenges. Defining a set of clear and robust rules to link related cases for all pathogens using WGS data, remains an active area of research (6–9). From a molecular evolution perspective, a “few” single-nucleotide differences can be interpreted as recent transmissions exposed to a common source, leading to the common practice of applying one or more genetic similarity thresholds to define epidemiological relatedness (7). However, the heterogeneous evolutionary rates among lineages and the variable nature of outbreak contexts (e.g., duration, extent, environmental pressure) render any choice of similarity thresholds prone to false positives and false negatives (6).

In practice, while genomic patterns are jointly analyzed with epidemiological data to guide investigations, the two data streams do not always converge on the same conclusion, which complicates interpretations and cluster detection. Polyclonal outbreaks are common scenarios that violate the assumption of clonality where strains or species with distinct genetic backgrounds accumulate in a common environment due to inadequate decontamination of food processing equipment or the manufacturing of meat mixture products, to name a few (10, 11). In such scenarios, detecting distinct subtypes from reported illnesses or applying stringent similarity thresholds could impede the establishment of linkages between polyclonal populations originating from a common source, potentially misguiding the investigators to assume the transmission of illnesses by independent pathways. On the other hand, the prolonged circulation of one or more clones in endemic regions can also render cluster attribution challenging, as the outbreak and background strains in endemics can frequently differ by as few as one single-nucleotide variant (SNV) (12). Investigators could interpret small SNV distances as probable linkages between epidemiologically unrelated strains, resulting in false attributions. To improve genotyping resolution, foodborne pathogen surveillance networks have increasingly shifted to genotyping bacteria at whole-genome scales (13, 14). The method, known as whole-genome multilocus sequence typing (wgMLST), characterizes the full complement of coding genes found in all strains of a given species (15). In contrast to the traditional approach of analyzing a restrictive set of highly conserved genes, known as core genome multilocus sequence typing (cgMLST), the inclusion of ancillary or accessory genes can reveal subpopulation divergence or selective sweeps driven by horizontal acquisitions of advantageous traits (16). One example is the rapid emergence of *Salmonella* ser. Infantis responsible for the 2008 Israel epidemic driven by a recent acquisition of a novel megaplasmid conferring increased resistance to environmental stress and antimicrobials (17). Recent comparative genomic studies have also attributed the evolution of bacterial accessory genes to zoonotic host specialization, niche adaptation, and geographical localization (18–22). In other words, microbial adaptation to unique environments and lifestyles can be commonly found imprinted in the ancillary component of bacterial genomes (20), which, in turn, could render accessory genome variations decisive for clustering illnesses caused by the dissemination of monoclonal or polyclonal populations from a common source.

However, the analysis of bacterial accessory genomes to inform epidemiological linkage remains controversial, with studies (23) questioning the generalizability of accessory genes for microbial subtyping and cautioning against the confounding effects of clustered polymorphisms in mobile genetic elements (MGEs). For example, extended transmission pathways involving multiple intermediate vehicles or migrations could drive increased rates of accessory gene exchange, thereby inflating the genetic distance between case isolates and the ancestral (source) clone (24). In this study, we sought to characterize the dynamics of bacterial accessory genomes in the context of *Salmonella* outbreaks to characterize the analytical value of MGEs for clustering outbreak cases. Owing to the increased accessibility to high-throughput sequencing technologies and open access to global genomic surveillance databases, such as GenomeTrakr and EnteroBase (25, 26), WGS data sets of well-characterized outbreaks are becoming increasingly available to train machine learning models from which genomic features representing molecular fingerprints of outbreak clones can be inferred. Here, we performed supervised machine learning on WGS data sets of 24 distinct outbreaks (239 isolates in total) caused by *Salmonella enterica* and identified an enrichment of markers in the *Salmonella* pan-genome that improved the clustering and discrimination of outbreak cases when benchmarked against gold standard methods (wgMLST and cgMLST).

## RESULTS

### *Salmonella* pan-genome graph

**Fig 1:**
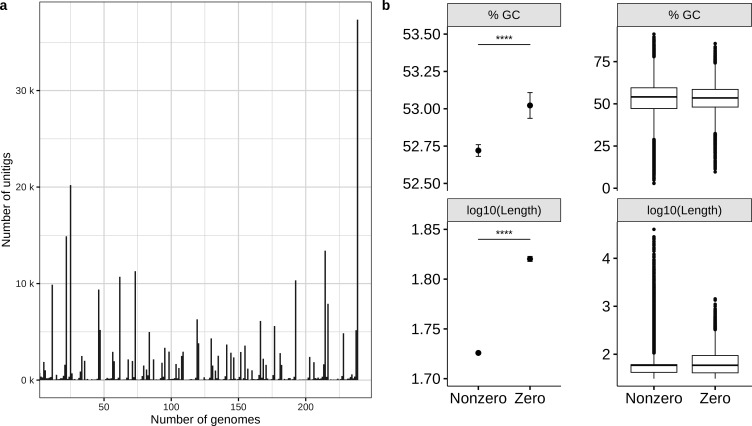
Comparing the sequence composition of variable and invariable unitigs in the training cdbG. (a) Identification of zero and nonzero variance unitigs in the training data set (*N* = 239) based on unitig incidence rates in the training genomes. Unitigs carried by 100% of the training genomes were classified as zero variance unitigs and filtered out prior to model fitting. (b) Comparing the mean difference (left) and distribution (right) of percentage GC content and log-transformed sequence length between zero and nonzero variance unitigs. The error bars represent the 95% CI. The mean percentage GC content and sequence length were compared between the two groups of unitigs using the Wilcoxon test.

**Fig 2:**
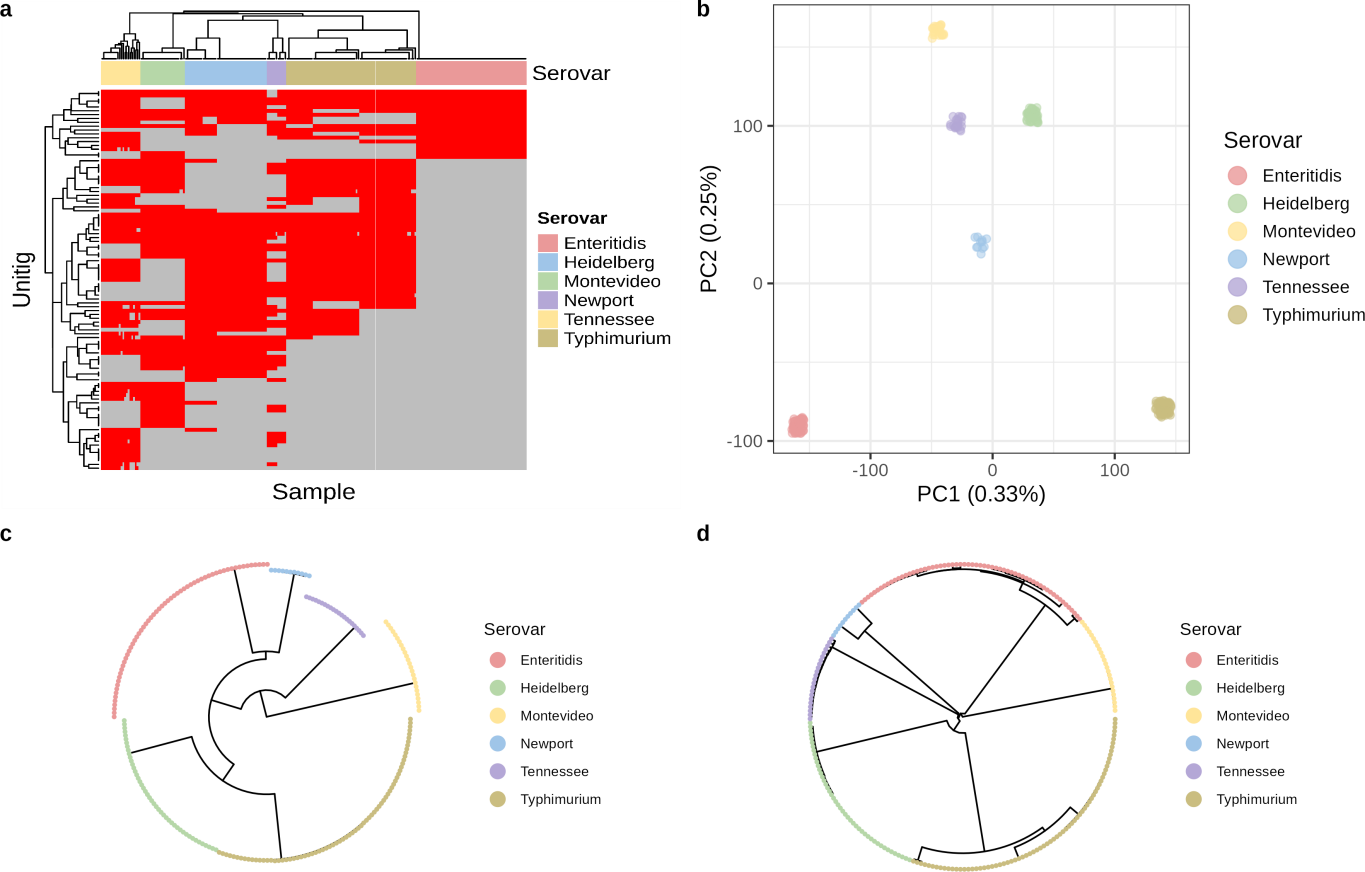
Population structure analysis of the training data set reveals evident population stratification by *Salmonella* serovars. (a) Hierarchical clustering of 10,000 randomly selected variable unitigs from the training pan-genome graph. (b) Dimensionality reduction of the binary profiles of variable unitigs (*N* = 260,219) from the training pan-genome graph by principal component analysis. The axes represent the first two principal components, together explaining 58% of the total variance (PC1: 33% + PC2: 25%) in the data. (c) Neighbor-joining tree of the training genomes (*N* = 239) constructed from cgMLST alleles called from a 3,000 loci cgMLST scheme. (d) Maximum likelihood tree constructed from the core genome SNV alignment of the training genomes (*N* = 239) using RAxML-NG.

Constructing a pan-genome graph from the outbreak training data set (Table SA1) yielded 297,563 unitigs, of which 12.5% (37,344) were present in every genome, i.e., variance of zero, called “invariable unitigs” hereafter (Fig. 1a). The combined sequence length of the invariable unitigs was 2.97 Mbps. In contrast, the combined length of variable presence unitigs (called “variable unitigs” hereafter) was five times longer (15.98 Mbps). The variable unitigs were frequently associated with more extreme percent GC content and sizes, suggesting differences in sequence composition between variable and invariable unitigs (Fig. 1b). Numerous sequence length outliers in the variable unitigs also fell within the size range of common bacterial MGEs (10–40 kbps), such as bacteriophages and plasmids (27, 28). Hierarchical clustering and principal component analysis (PCA) of the unitig profiles (binary vectors of unitig presence/absence) revealed evident population stratification (Fig. 2a and b), suggesting the need for population structure correction during model training. The observation of a strong population structure was concordant with the phylogenies constructed from the same data set by cgMLST and core genome SNV analysis (Fig. 2c and d).

### Outbreak marker selection and model performance

**Fig 3:**
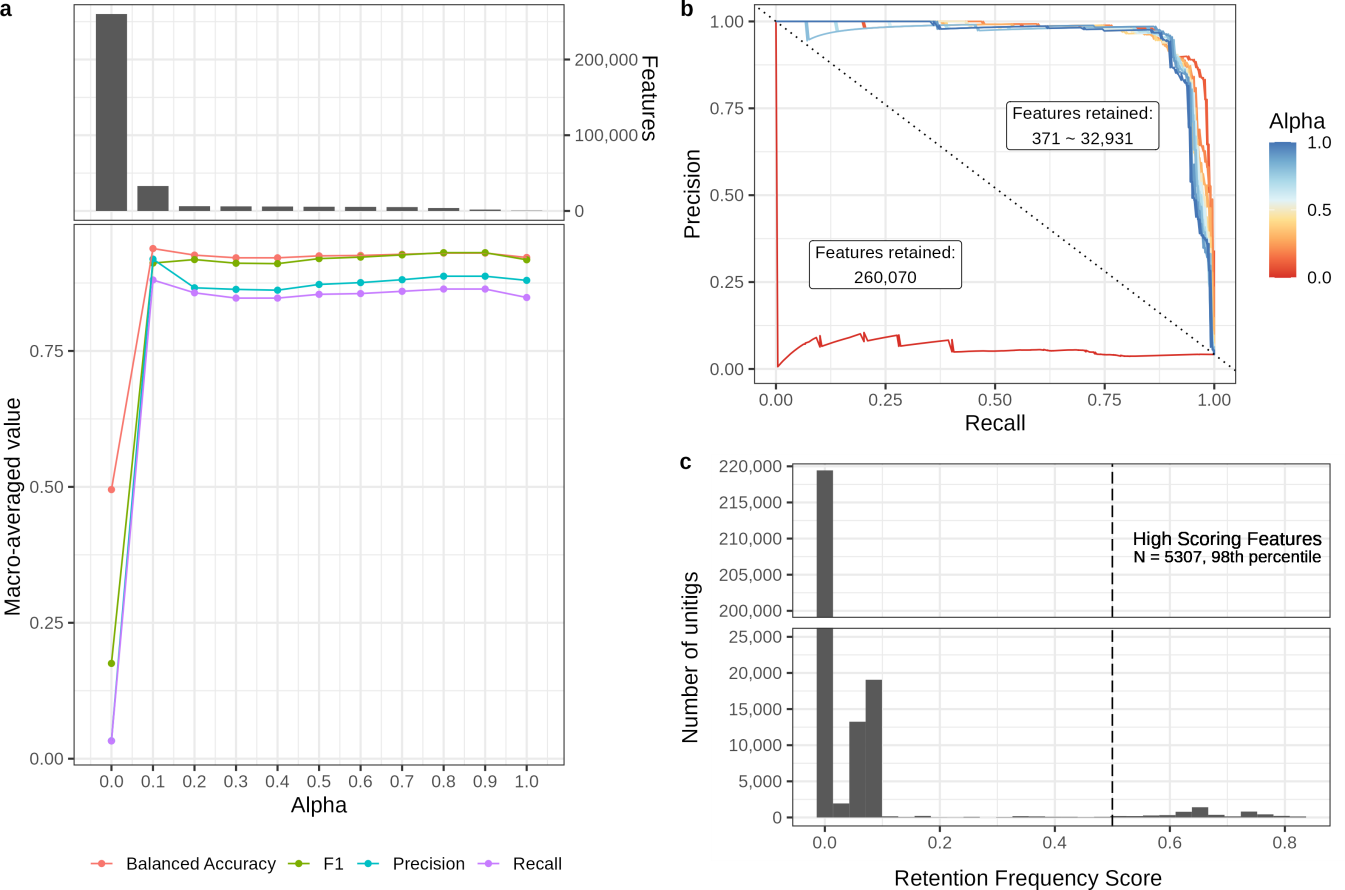
The effects of alpha hyperparameter tuning in elastic net regularization on model performance and complexity. (a) Comparison of macro-averaged model performance scores across a spectrum of alpha values. The top subplot shows the number of features retained in regularized models with respect to alpha. (b) Precision-recall curves segregated by alpha values. Quantifying areas under the precision-recall curve provides an overall assessment of model performance in which a smaller area under the curve suggests worse classification performance. (c) Distribution of retention frequency score (RFS). A low RFS value indicates a low retention rate of a given feature in good performance models. A cutoff of RFS = 0.5 was employed to select a narrow subset of 5,307 features to represent the key genomic signatures of the training outbreaks.

When tuning the alpha hyperparameter (L1/L2 ratio) in elastic net regularization, we observed the expected trend of increased model parsimony with larger alpha values (Fig. 3a). Increasing model parsimony concurrently led to significantly improved model predictions on cross-validation (CV) test sets (Fig. 3b). The highest macro-averaged F1 score and balanced accuracy were observed at alpha = 0.1, indicating that model performance did not increase monotonically with alpha. Reduced performance was observed at the extremities of the spectrum, with the sparsest model (alpha = 1.0) likely underfitting and the most complex model (alpha = 0) likely overfitting (Fig. 3a). While comparing the model performance at classifying the individual outbreaks, we observed a select number of outbreaks that our models consistently struggled to classify, irrespective of alpha values (Fig. SA1). Interestingly, we observed an inverse correlation between model performance and the maximum pairwise cgMLST distance (*D*) of an outbreak cluster, which also covaried with outbreak duration. We observed poor classification performance on two different outbreaks associated with ser. Tennessee, both of which had *D* > 300 and estimated durations of longer than 6 months (Fig. SA1). Although our models robustly classified outbreaks that were highly clonal (*D* < 50) and short-lived (estimated duration ≤1 month), there did not exist a single distance threshold or outbreak period cutoff that could perfectly explain the polar model performance in different outbreak scenarios.

Instead of selecting important features based on the best-performing model, we devised an agglomerative score that integrated information from all model fits to rank the relative importance of each feature in the training data. Based on the rationale that the retention of irrelevant features and the removal of important predictors increase model errors, we used performance-weighted frequencies of feature retention across CV folds as a proxy for feature importance. The resulting measure, named “retention frequency score” (RFS), exhibited a right-skewed distribution with >90% of the unitigs having an RFS less than 0.1, indicating a low retention rate in good performance models (Fig. 3c). We chose an RFS cutoff of 0.5 to select a narrow subset of 5,706 unitigs that constituted the key genomic signatures of the training outbreaks.

### Annotation and functional enrichment of outbreak markers

**Fig 4:**
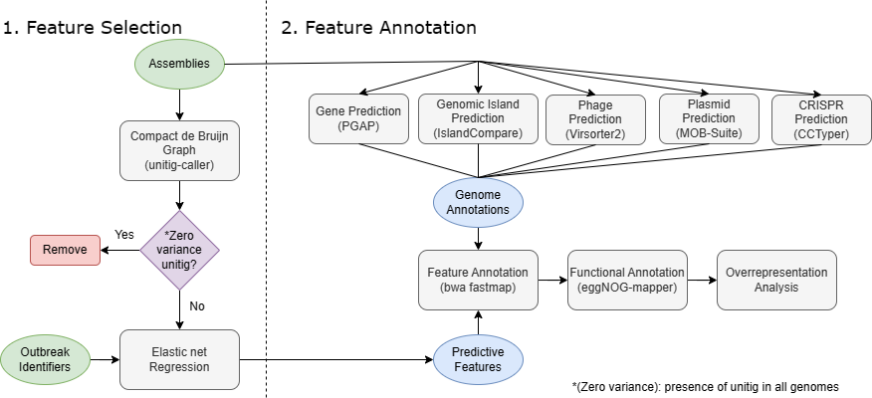
Overview of the analysis workflow. In the first phase of the workflow, training outbreak genome assemblies (.FASTA) were downloaded from GenBank to construct a single compacted de Bruijn graph (cdBG) representing the pan-genome of the collection of training genomes. The unitig sequences were extracted from the training cdBG and queried against the cdBG to construct a binary matrix encoding the presence and absence of unitigs in the training data set. Prior to model training, uninformative features, i.e., zero variance, were removed. The training data set was split into cross-validation folds to assess the performance of regularized multivariate models at different alpha values, a hyperparameter in elastic net regularization. Feature selection was conducted based on an agglomerative score that measured the feature retention frequency in a collection of model fits as a proxy for feature importance. In the second phase, the sequences of the selected features were mapped back to the training genome assemblies to pinpoint the genomic origins of the unitigs. Genome annotation tools, including numerous mobile genetic element prediction software, enabled the annotation of the selected unitigs in coding or noncoding genes and chromosomal or extrachromosomal regions. The protein products encoded by the coding regions intersecting the selected unitigs were searched against the eggNOG database to categorize gene function. Lastly, the observed frequency of each functional category was subjected to overrepresentation analysis using the hypergeometric test to discover biological function or pathway enrichment.

**Fig 5:**
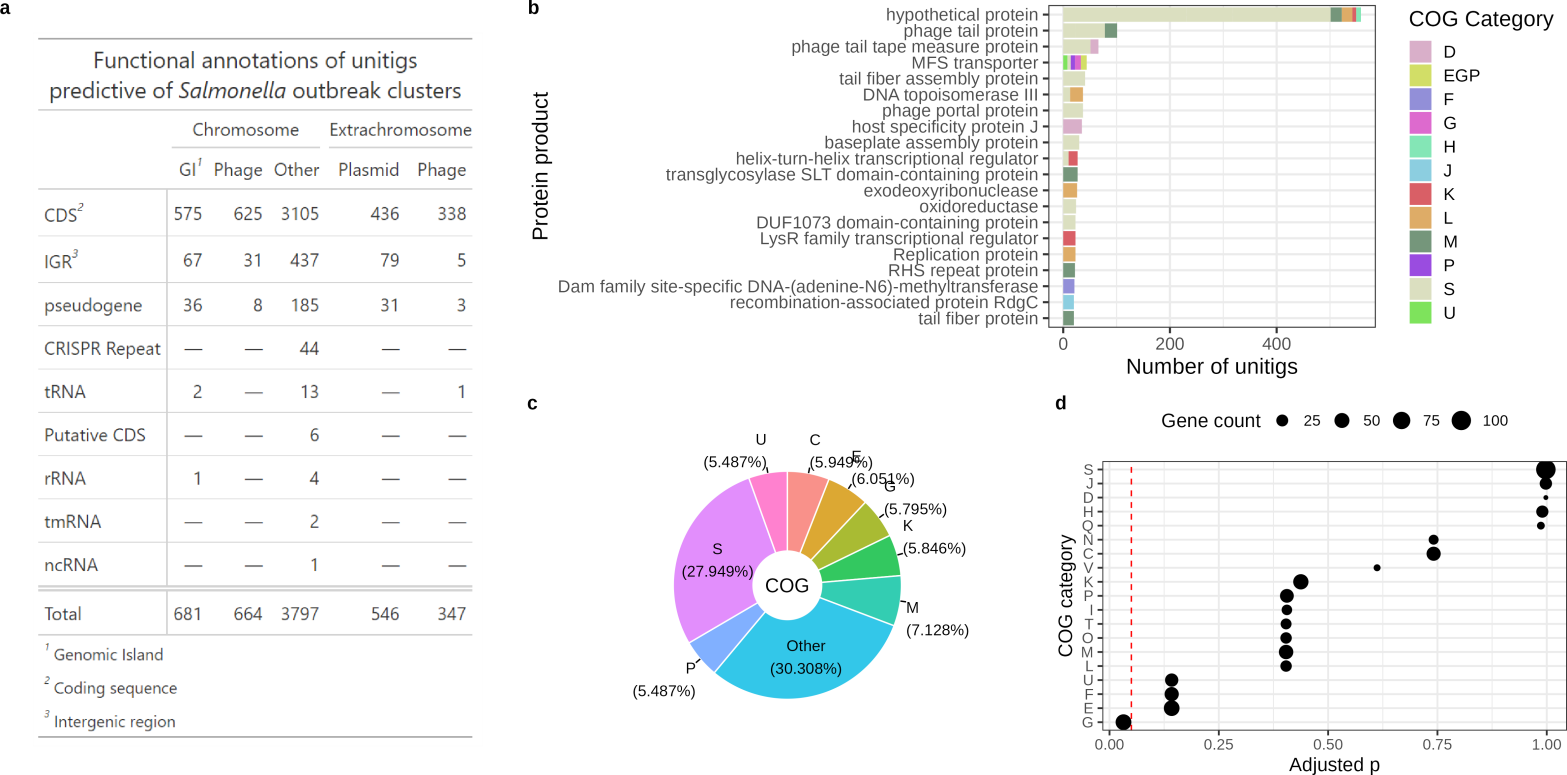
Annotation of important predictors (unitigs) of *Salmonella* outbreaks. (a) Breakdown of outbreak signatures in chromosomal and extrachromosomal regions spanning coding and noncoding sequences. Note that large unitigs spanning multiple functional elements can be assigned to multiple classes in the table. (b) Breakdown of the protein products encoded by coding genes carrying outbreak signatures. (c) COG assignment of the protein products encoded by coding genes carrying outbreak signatures. (d) Overrepresentation analysis of the COG categories assigned to coding genes mapped by important unitigs reveals enrichment of outbreak signatures in categories G and U.

To interpret the outbreak genomic signatures in a biological context, the functions of the model-selected unitigs (MSUs) were annotated based on their genomic origins (Fig. 4). Approximately 84% of the MSUs mapped to coding genes (Fig. 5a). Of the 8,634 coding genes in the training pan-genome predicted by panX (29), the MSUs mapped to 20% (1,954/8,634) of the total coding genes. The majority of the mapped genes (1,081/1,954, 55.3%) were present in 99% of the training genomes. Greater than 20% of the MSUs mapped to genes that encode for functionally unknown products (Fig. 5b and c). Functional enrichment analysis detected the enriched presence of MSUs in core genes belonging to two Clusters of Orthologous Group (COG) categories: G and U (Fig. 5d). Enriched category G (Carbohydrate transport and metabolism) genes included important regulators of sugar metabolism (e.g., malP, glgX, glgP) implicated in sustaining bacterial viability under harsh conditions when carbon sources are scarce (30, 31). Category U (Intracellular trafficking, secretion, and vesicular transport) genes, commonly situated in bacterial genomic islands (GIs) (32, 33), encoded for multiple membrane transport protein families regulating the fluxes of a diverse range of molecules from macromolecules, such as proteins (e.g., secABDEF) and polysaccharides (e.g., celB) to small solutes, such as antimicrobial compounds (e.g., mdtBHIJL), amino acids (e.g., yifK, yjeM, rhtBC), and metal ions (e.g., exbB). Moreover, repetitive elements, such as clustered regularly interspaced short palindromic repeats (CRISPR), Ig-like repeat domain proteins, and rearrangement hotspot repeat proteins, were also identified to carry cluster-specific features. Annotation of the training genomes using a suite of *in silico* MGE prediction tools (Fig. 4) identified ~37% of the MSUs to localize in numerous chromosomal and extrachromosomal MGEs, including GIs, prophages, free phages, and plasmids (Fig. 5a).

### Explaining the importance of MGEs for cluster classification

Explaining the importance and contribution of the identified markers is an equally important part of marker discovery. As the original intention of our study was to characterize the analytical value of MGEs in *Salmonella* outbreak clustering, we conducted one case study that characterized the mutational drift of MGEs to explain their retention in our regularized models. Despite the conservation of the CRISPR-Cas system in *Salmonella*, we decided to also include the CRISPR array as an MGE of interest due to its natural maintenance of exogenous spacer sequences and the recent attention to CRISPR arrays for bacterial typing (34, 35).

**Fig 6:**
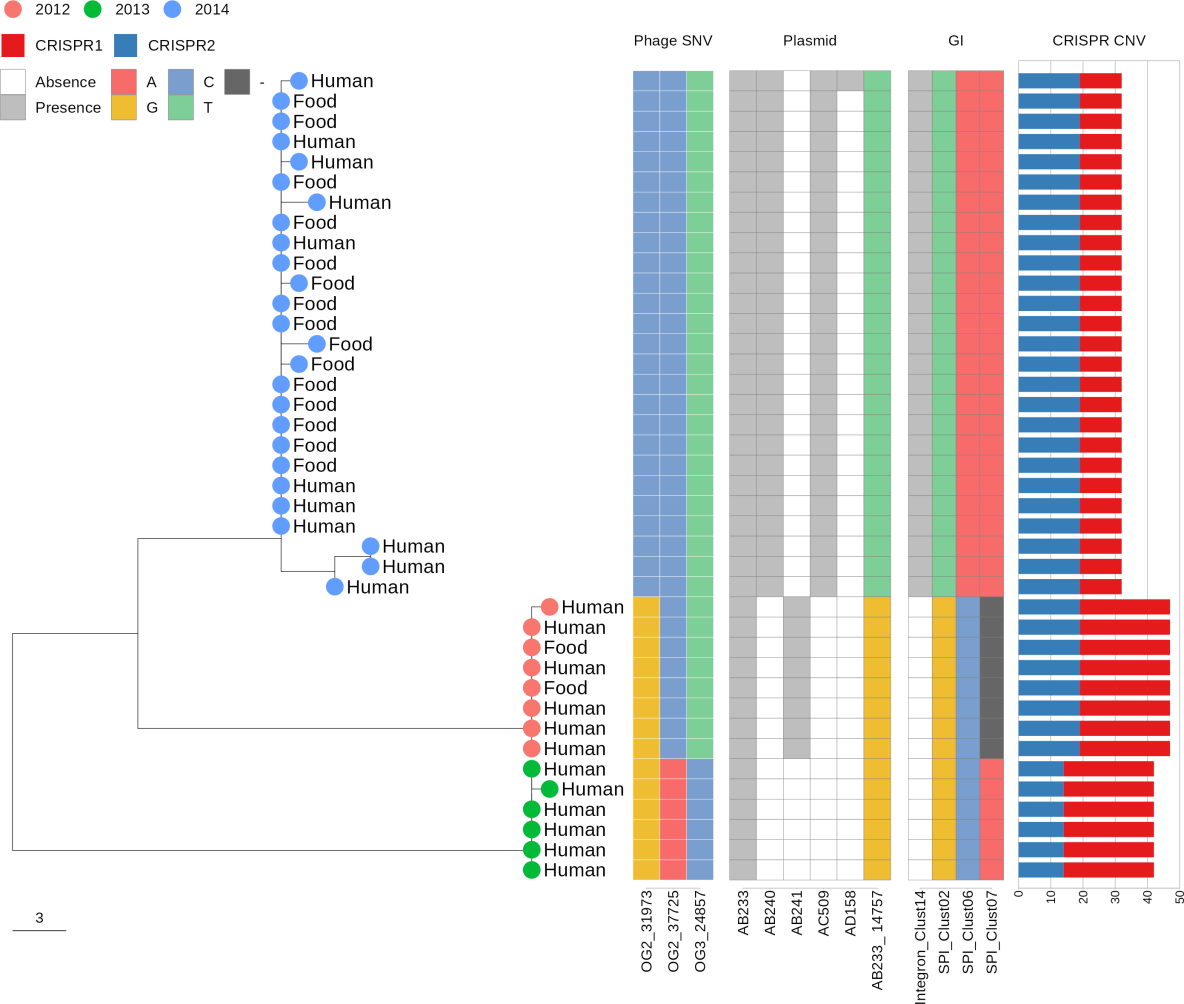
Midpoint-rooted cgMLST phylogeny of three point-source foodborne outbreaks in the Province of Quebec, Canada, caused by *Salmonella* ser. Heidelberg. The phage SNV columns represent polymorphic sites detected in two phage orthologous groups (OGs) labeled OG1 and OG2. Sequence coordinates of the polymorphic sites are appended after the phage OG labels. The plasmid columns represent plasmid cluster identifiers assigned by MOB-suite. For polymorphic plasmids, e.g., Cluster AB233, sequence coordinates of the polymorphic sites are appended after the plasmid cluster identifiers. The genomic island (GI) columns represent cluster identifiers assigned by IslandCompare and categorized as *Salmonella* pathogenicity island (SPI) or integron based on the presence of hallmark genes. The CRISPR copy number variation (CNV) barplot illustrates the number of spacer-repeat units detected in CRISPR locus 1 (red) and locus 2 (blue) by CRISPRCasTyper. Note: six outbreak genomes (GCA_001690005.1, GCA_001689935.1, GCA_001692635.1, GCA_001692535.1, GCA_001692655.1, GCA_001690035.1) were removed from the analysis due to evidence of misassembly.

Our case study involved three ser. Heidelberg foodborne outbreaks in the Province of Quebec, Canada, each of which occurred in a different year between 2012 and 2014 (36). Epidemiological follow-ups classified the three outbreaks as point-source outbreaks caused by *Salmonella*-contaminated foods served from a common source (36). Food and human isolates of the three outbreaks were reported to exhibit indistinguishable PFGE patterns, which invited the application of WGS to resolve the case clusters. Concordant with the SNV analysis conducted by Bekal et al. (36), we identified MGE signatures sufficient to explain the segregation of the three foodborne outbreaks (Fig. 6).

In every genome, we detected the presence of phage sequences belonging to three orthologous groups (OGs), two of which were polymorphic (Fig. SA2). A total of three single-nucleotide substitutions were detected in the phage sequences. The genotypes of the three polymorphic sites formed unique haplotypes that were sufficient to differentiate the three outbreaks (Fig. 6). Two of the three substitutions caused nonsynonymous mutations in two different coding genes encoding for DUF550 domain protein (GenBank accession: HAE5201317.1) and phage tail protein (GenBank accession: HAE5201269.1), while the third substitution occurred in an intergenic region.

Comparing the plasmid sequences revealed the unique presence of two mobilizable plasmids in every 2014 outbreak isolate. The plasmid prediction tool, MOB-suite (37), assigned the two plasmids, ColRNAI (6.6 kbps) and ColpVC (2 kbps) to plasmid clusters AC509 and AB240, respectively. The presence of another 2 kbps ColpVC plasmid assigned to cluster A241 was a distinguishing feature for the 2012 outbreak isolates. IncX1 plasmid (37 kbps), a feature common to all Heidelberg isolates in the case study, carried polymorphisms that uniquely identified the 2014 outbreak isolates (Fig. 6).

Based on previous findings that the lengths of CRISPR arrays frequently varied across *Salmonella* lineages (38, 39), we compared the number of CRISPR spacer-repeat units (SRUs) in the two CRISPR loci commonly present in *Salmonella*. As expected, a characteristic number of SRUs was found in each outbreak. Interestingly, isolates from the earlier 2012 outbreak carried the highest number of SRUs, while the more recent 2014 outbreak had the least number of SRUs (Fig. 6).

Among the ser. Heidelberg outbreaks, we identified 14 GIs, most of which were identified as *Salmonella* pathogenicity islands (SPIs). Two GIs were prophages, the sequences of which overlapped two of the three phage OGs mentioned above. Of the non-phage GIs, four islands (three SPIs and one integron) were polymorphic. Two SPIs formed haplotypes unique to each outbreak with single-nucleotide substitutions identified in two genes: spaL (ATP synthase) and puuP (Putrescine importer) (Fig. 6). Evidence of recombination was detected in the third SPI due to the occurrence of >100 SNVs in the span of 2 kbps. PCA of the SNV matrix from the third SPI revealed that the 2012 and 2013 isolates shared a common haplotype (Fig. SA3). The final polymorphic GI encoded key hallmarks of integrons: endonuclease and integrase. Embedded in between the two enzymes were numerous tandem gene cassettes that expressed glycosyltransferases and transporter proteins. Although the comparison of the integron sequences resulted in no detectable polymorphisms, the variable presence of the integron among the outbreak genomes suggested that large indels (>50 bp) can serve as informative markers for cluster attribution (Fig. 6).

Inspired by alignment-free tree algorithms that have enabled the rapid clustering of related sequences by comparing reduced representations of genomic sequences (40, 41), we sought to explore the feasibility of representing isolate relatedness in a tree structure by adapting unitig profile similarities as a proxy for genetic similarity. We measured the differences between two unitig profiles in hamming distances, which could be rapidly clustered using the neighbor-joining (NJ) algorithm. Discriminating between unitigs derived from the various MGEs subsequently enabled the construction of dendrograms to depict clustering patterns among outbreak isolates based on MGE sequence variations. In line with cgMLST and wgMLST, the dendrograms constructed from the individual MGEs also clustered the three Heidelberg outbreaks into distinguished clades (Fig. SA4).

### Confounding effects of assembly contiguity

**Fig 7:**
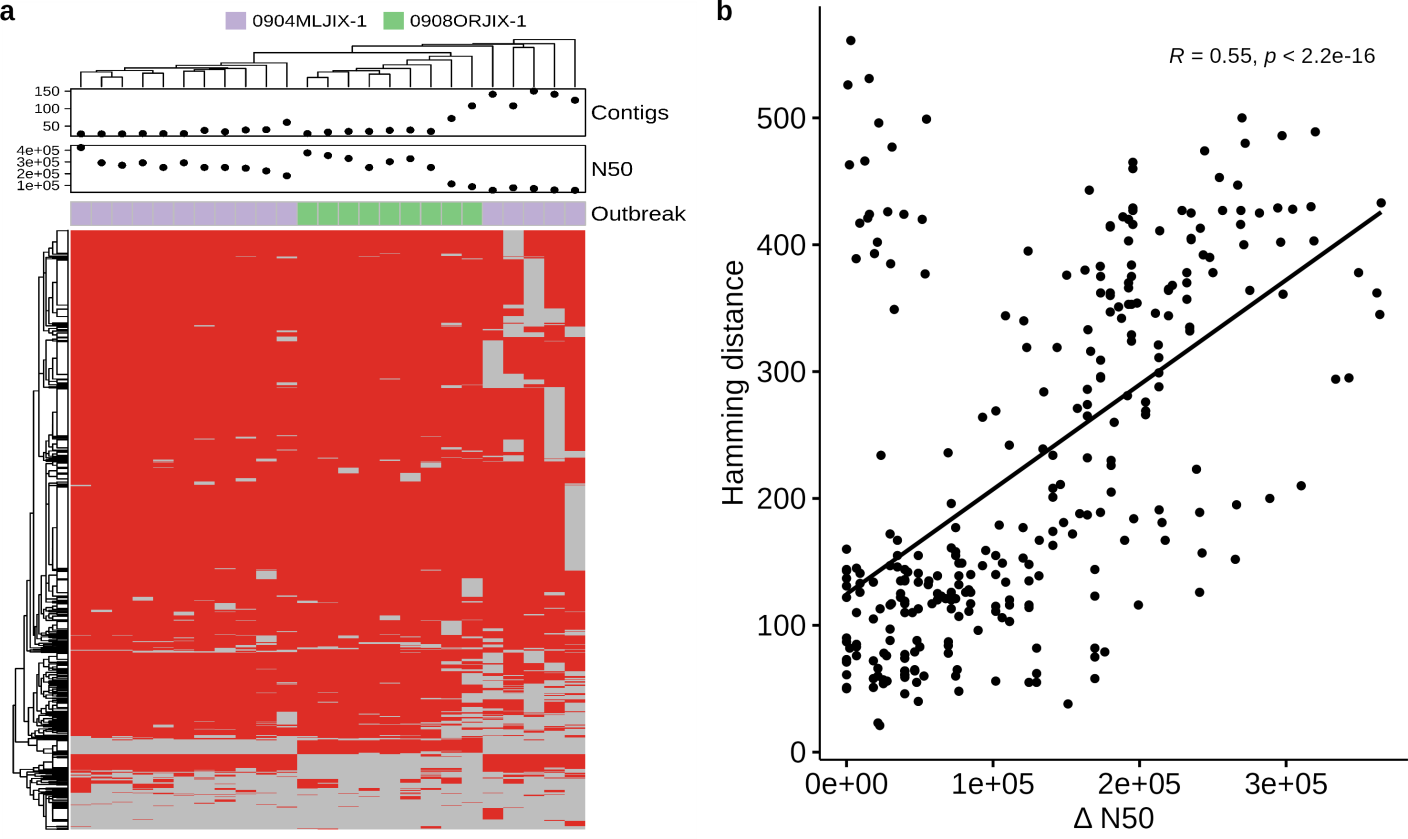
Potential confounding effects of inferring genetic similarity by unitig profile distance. (a) Hierarchical clustering of unitig profiles of two *Salmonella* ser. Montevideo outbreaks in the training data set. The subplots above the main heatmap compare the number of contigs and N50 score of the ser. Montevideo genomes. (b) Pairwise unitig profile similarity (measured in hamming distance) computed between genomes of the two *Salmonella* ser. Montevideo outbreaks as a function of N50 score differences. Quantifying the degree of linear correlation between the two dimensions yielded a pearson correlation coefficient (*R*) of 0.55.

While unitig profiles were shown to produce tree topologies concordant with epidemiological data from our case study, we also encountered examples of topological discordance that could be partially explained by sequence data quality. We attributed the topological discrepancies to the emergence of unitigs that likely represented artifacts of genome assembly errors and gaps. In two ser. Montevideo outbreaks that produced genomes of variable N50 values (20–200 kbp), we identified an array of unitigs that were exclusively present or absent in highly fragmented assemblies (N50 < 100 kbp). When the isolates were hierarchically clustered by unitig profiles, we observed segregation by assembly contiguity in the resulting dendrogram (Fig. 7a). Further analysis revealed collinearity between unitig distance and assembly contiguity, suggesting stringent control of assembly quality is needed to mitigate the confounding effects of assembly artifacts on the genetic distances inferred from unitig profiles (Fig. 7b).

### Benchmarking MGE clustering performance using an unseen data set

To validate the efficacy and generalizability of genotyping MGEs to cluster outbreak cases, we benchmarked MGE clustering against genomic subtyping methods preferred by PulseNet (3), namely, cgMLST and wgMLST. Our benchmark leveraged a measure called “monophyletic rate” to quantify clustering performance on two data sets consisting of the original training data set and an unseen validation data set of 34 outbreaks. The basis of this measure is founded on the expectation that an optimal outbreak clustering method should group epidemiologically related cases in closer proximity and impose greater separation between unrelated cases. Hence, we rationalized that clustering performance could be quantified by the monophyletic clustering of outbreak isolates in NJ trees. Formally, monophyletic rate is defined as the fraction of the total training or validation outbreaks whose isolates formed monophyletic groups in rooted NJ trees. To evaluate whether unitigs can be extended to analyze core genome differences, our benchmarked methods additionally included ggcaller, a pan-genome tool that annotates core and accessory genes directly from cdBGs (42). Similar to the clustering of MGE unitigs performed in our case study, the core genome annotated by ggcaller can also be vectorized as unitig profiles and clustered.

**Fig 8:**
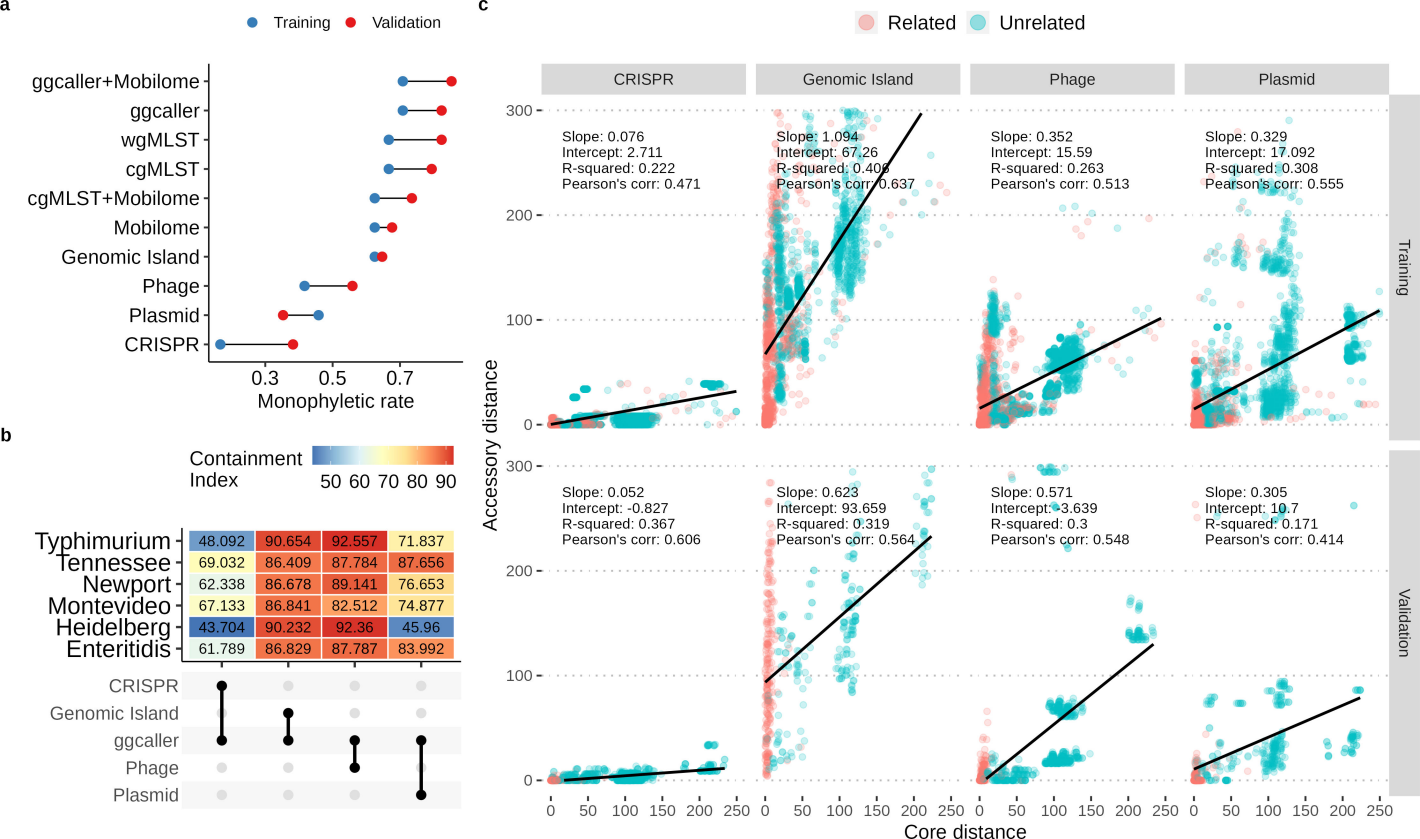
Benchmarking the performance of mobile genetic element (MGE) and ggcaller clustering against gold standard subtyping approaches using training and validation data sets. (a) Comparing the monophyletic rate of dendrograms constructed from different input sources. Two MLST schema containing 3,000 and 8,558 loci were used to call cgMLST and wgMLST alleles, respectively. The cgMLST and wgMLST trees were constructed by transforming the allelic profiles into distance matrices and clustered using the neighbor-joining (NJ) algorithm. The MGE and ggcaller dendrograms were constructed by building compacted de Bruijn graphs for each serovar in the training or validation data set and identifying unitigs that mapped to MGE or ggcaller contigs. The binary profiles of the selected unitigs were clustered by the NJ algorithm to construct MGE and ggcaller dendrograms. The “mobilome” refers to the combined clustering of genotypes derived from all MGEs (CRISPR, genomic island, phage, plasmid). Monophyletic clustering of a vector of tips in a given tree was assessed using MonoPhylo. (b) Quantifying the containment of MGE unitigs in the core genome unitigs identified by ggcaller across training outbreaks stratified by serovars. (c) Correlation between pairwise accessory distance (measured in MGE unitig distance) and pairwise core distance (measured in cgMLST distance). Pairwise comparisons between epidemiologically related and unrelated isolates are colored red and blue, respectively. Linear models were fitted to the data to estimate the rate of change in accessory distance per unit change in core distance.

The dendrograms constructed from MGEs exhibited variable performance in clustering the training outbreaks. Of the MGE dendrograms, GI produced the highest monophyletic rate, and CRISPR produced the lowest monophyletic rate (Fig. 8a). The diminished performance of MGE clustering compared to gene-by-gene typing could be attributed to the conservative nature of MGEs in the context of *Salmonella* outbreaks, as the core genomes of unrelated cases frequently exhibited greater variability than the accessory elements investigated, except for GIs (Fig. 8c). The elevated performance of GIs in respect to other MGEs could be explained by the directly proportional relationship between GI and core genome distances inferred from linear modeling (Fig. 8c).

In scenarios where the core genomes of unrelated isolates were indistinguishable, we found the analysis of MGEs to benefit typing resolution. Two ser. Typhimurium outbreaks (0811MLJPX-1c and 0811SDCJPX-1c) were found indistinguishable by cgMLST but segregated into monophyletic groups when clustered by plasmid sequences or wgMLST (Fig. SA5). In another case, the core genome of one ser. Enteritidis human isolate from an egg-related outbreak (O) in Georgia, USA, 2005, was indistinguishable from human fecal isolates collected from another egg-related outbreak (MN-3) in Minnesota, USA, 2001. Analyzing accessory variations in the pan-genome, including CRISPR arrays and genomic islands, led to the discovery of outbreak-specific signatures that enabled the discrimination of the two egg-related outbreaks (Fig. SA6). The best clustering performance was achieved by ggcaller dendrograms, surpassing cgMLST and wgMLST by a small margin (Fig. 8a). In response to the superior performance displayed by ggcaller dendrograms that only accounted for core genome features, we subsequently investigated the possibility of improving its performance via the inclusion of MGE features, analogous to wgMLST. Surprisingly, the effects were negligible (Fig. 8a). Analyzing set intersections between core genome and MGE unitigs later revealed significant redundancy, which explained the impotence of integrating MGE features (Fig. 8b).

The clustering performance of MGEs and ggcaller generalized well to the validation data set unseen in model training. The relative ranking of each method remained fixed except for plasmids and ggcaller that ranked lower in the exclusion of MGEs (Fig. 8a). In both training and validation data sets, numerous outbreaks did not form monophyletic groups in any dendrograms (Fig. SA7 and SA8). These poorly clustered outbreaks were associated with a larger *D*, which would explain the lack of common genotypes shared between the related isolates. However, a nut-related outbreak (1109NYJEG-2) associated with ser. Enteritidis was a visible outlier (Fig. SA8). The CRISPR dendrogram revealed that the nut isolates could be linked by CRISPR typing in spite of *D* > 60 (Fig. SA6).

## DISCUSSION

The availability of pathogen WGS data generated by public health, clinical diagnostics and research has attracted significant interest in the application of machine learning to untangle the complex patterns and relationships underpinning the spread and emergence of infectious diseases. Designing targeted intervention strategies to mitigate and control disease transmission relies on the timely identification of relevant cases to hypothesize epidemiological links between the suspected cases and putative source populations of zoonotic, food or environmental origin (43–45). In this study, we demonstrated the feasibility of training machine learning models capable of accurately classifying clusters of *Salmonella* isolates sampled from historical outbreaks using WGS data. The inferences drawn from our regularized models illuminated genomic regions likely to harbor outbreak distinguishing features either caused by random mutations or important niche adaptations. The enrichment of outbreak markers in specific functional elements (e.g., MGEs, carbohydrate metabolism, solute uptake, and secretion) suggests that the molecular evolution of epidemic clones can be directed by environmental factors, such as the local microbiome, nutrient availability, or stress. This is concordant with previous studies that have emphasized the important contribution of metabolic genes and MGEs in explaining the preferred localization of *Salmonella* in specific hosts and geographical regions (18, 46–48). The pronounced proportion of outbreak markers mapping to MGEs supported our hypothesis that molecular fingerprints of outbreak clones can be found imprinted in the ancillary component of bacterial genomes. To reinforce our argument, we documented various scenarios in which different classes of MGEs independently produced epidemiological trends convergent with core genome analysis (Fig. SA7 and SA8). More importantly, we identified numerous scenarios in which accessory genome variations were decisive for clustering foodborne outbreak cases either by surpassing cgMLST resolution (Fig. SA5) or in one scenario, linking mixed populations (Fig. SA6). The efficacy of accessory variations to resolve closely related strains suggested that while the accessory regions are considered hypervariable in respect to the core genome and subjected to numerous mechanisms of horizontal gene transfer, many MGEs frequently remain stable during clonal expansion. The ser. Heidelberg outbreaks from our case study exemplified the stable maintenance of recently acquired accessory variations that were characteristic to each outbreak.

The usage of unitigs to encode a broad range of genetic variants in a population of bacterial genomes was pivotal in associating accessory genotypes to outbreak clusters. In contrast to classical genome-wide association studies (GWAS) that restricted the scope of hypothesis testing to single nucleotide polymorphisms, unitigs offer the capacity to additionally test phenotypic associations on large indels, e.g., gene gain/loss and structural variants, e.g., copy number variations. The versatility of the method consequently enabled the illumination of informative features in accessory and repetitive regions often masked or filtered by standard sequence typing methods (49–51). To explore the application of unitigs beyond bacterial GWAS and facilitate the knowledge translation of our findings, we devised a distance-based tree algorithm exploiting the sensitivity of unitigs to pan-genome variations to cluster sequences and construct dendrograms from bacterial core genomes, MGEs, or the combination of both. From benchmarking against gene-by-gene typing, our method proved highly accurate in constructing tree topologies consistent with epidemiological data, especially during the joint clustering of the mobilome with the core genome inferred by ggcaller. The efficacy of clustering unitigs is threefold: (i) genotyping beyond coding regions, (ii) unbiased by reference sequences or schema, and (iii) preventing the inflation of genetic distances by treating large horizontally acquired variants (e.g., an entire plasmid) as one unit of variation. While wgMLST was devised to compare genome-wide variations, the stagnant nature of MLST schema, i.e., typing a fixed set of genes hinders the discovery of potentially informative alleles on novel accessory genes. Likewise, cgMLST is restricted to typing core genes inferred from a collection of references that in all likelihood share a different set of core genes from the genomes under investigation. Sequence variations in any given collection of genomes can be encoded as branching paths and node colors in cdBGs, producing a data structure that can be queried to investigate population structure and microevolution events. With the help of compatible graph search algorithms (42, 52, 53), regions in any cdBGs can be structurally annotated to enable sequence typing based on a dynamic set of core or accessory loci specific to the genomes under investigation. The unbiased and reference-free modeling of genetic variations will prove valuable in enhancing the resolution of whole-genome typing and the molecular surveillance of MGE-driven propagation of clonal or mixed populations.

Beyond the scope of this study, it would be interesting to characterize the functions of the cargo genes carried by each MGE to correlate the functional impacts of MGEs on bacterial fitness to their microevolutionary trajectories. While we did not examine the biological functions of individual MGEs herein, we postulate that functional differences could have contributed to the variance in the observed mutation rates among MGEs, leading to the variable efficacy of different MGEs in producing monophyletic clusters. Previous studies (54–56) have reported that the carriage of genes in MGEs that confer a fitness advantage to the bacterial host have the tenacity to stabilize within the host genome. Conversely, the introduction of neutral or mildly deleterious accessory genes that create an imbalance between cost and benefit could accelerate the removal of the MGE or even the host genome from the population by the process of purifying selection (57, 58). It has also been reported that the transient nature of MGEs could be further compounded by the context-dependent behavior of molecular evolution that renders the presence of an MGE beneficial in certain contexts but parasitic in others (59). Progressing our understanding of the factors governing the evolution of MGEs will drive the development of improved algorithms to model the molecular evolution of accessory sequences and enable a more precise selection of appropriate markers for outbreak clustering.

One glaring limitation of our study is our decision to exclude background strains. Our benchmark did not rigorously simulate the scale of comparisons routinely performed in surveillance settings (60–63). As a result, it is plausible that we have overestimated the number of outbreak markers due to the scarce level of noise in the study data set. Furthermore, the extent of homoplasy, i.e., independent acquisition of the same MGE among unrelated *Salmonella* lineages, was not critically evaluated in our benchmark, as the training and validation data sets were segregated into serovar clusters from which the dendrograms were independently constructed. We have also approached the cluster classification problem solely from a genomics perspective. We did not account for potential interaction effects between pathogen genetics and epidemiological factors. For example, the predictive value and mutation rate of a functional element could be influenced by outbreak origins, intermediaries, and geographical range. Susvitasari et al. recently applied pairwise logistic regression to predict outbreak clusters of *Mycobacterium tuberculosis* cases using demographic data and found human host nationality and spatial distance to be important determinants of epidemiological linkage (64). Considering that demographic and epidemiological parameters can contribute to the likelihood of linkage, the inclusion of additional covariates to stratify training outbreaks by epidemiological scenarios may improve model fit and untangle important interaction effects.

Although the massive volumes of pathogen WGS data generated by research, surveillance and clinical diagnostics are readily accessible from online repositories, e.g., NCBI databases, the quality of the metadata linked to these sequence data is largely lacklustre (65). While investigating the current state of the research data ecosystem in public domains goes beyond the scope of this study, we briefly described the inefficiencies we encountered during data collation (see Materials and Methods, “Study data set”) as a means to provide some anecdotal evidence to support the inadequacies of existing systems at storing, sharing, encoding, and finding research data. By highlighting the current laborious nature of data collection and standardization from primary articles, we sought to inspire movements among the broader community to advocate for data governance frameworks that promote the reusability and harmonization of primary data for research. Relative to sequence data, the quality of sample attributes in databases is dismal despite the information being a critical component to the interpretation of sequence data. As a result, it remains a non-trivial exercise to compile data from independent sequencing projects at a scale and quality level necessary to train generalizable models from which meaningful biological insights can be deduced. With the increasing production of data to drive knowledge generation, reducing the existing burdens of synthesizing data across independent studies is paramount to promote greater data reuse and embrace the full benefits of open data and open science. By building a more connected data ecosystem and supporting collaborative learning, we can expedite scientific discoveries and improve the productivity of data-driven research, leading to more innovative approaches to exploit pathogen WGS data.

## MATERIALS AND METHODS

### Method overview

To quantify the performance of bacterial accessory sequences at predicting epidemiological relatedness in the context of *Salmonella* outbreaks, we approached the outbreak cluster classification problem solely from a genomics perspective by training interpretable machine learning models on genome assemblies of previously reported outbreak strains. To account for the diverse range of genetic events and the plasticity of bacterial genomic sequences, we opted for a reference-free and alignment-free representation of genomic variations called compacted de Bruijn graphs (Fig. 4). Any degree of sequence variations forms alternate branching paths and introduces additional nodes in cdBGs called “unitigs” (66), the presence and absence of which can be encoded as numerical predictors in the mapping function of machine learning models. Ultimately, we are interested in characterizing outbreak markers, defined as any locus found in the bacterial pan-genome that can inform cluster attribution by genotyping the locus. In other words, we aimed to identify a parsimonious set of genomic markers that can explain and accurately predict the segregation of all *Salmonella* outbreak clusters. Logically, the ideal candidates would correspond to loci that carry comparable genotypes between epidemiologically related isolates (same outbreak) and divergent genotypes among unrelated isolates (different outbreaks).

Inferring the relative variance of an outcome explained by a set of parameters and covariates is a common machine learning task. However, training machine learning models on high-dimensional data sets is prone to overfitting due to highly variable feature importance (67). To prevent overfitting and produce models that generalize well to unseen (test) data, we imposed penalty terms that shrank the effect sizes of explanatory variables in the cost function of regression models—a process known as regularization. The outcome of regularization is a sparser model from which important features can be inferred and annotated to elucidate putative outbreak markers. In the absence of multiple testing burden, regularized linear models have been shown to achieve greater statistical power compared to classical statistical testing of association (67–70), rendering it a robust approach for GWAS where the number of features frequently exceeds the sample size.

Given that unseen outbreak strains are unlikely to carry the exact same set of genomic features that were used to train our regularized models, it is inapplicable to utilize our models to classify novel variants. However, we reasoned that the predictive power of an individual locus or the functional class of a locus (e.g., CRISPR, phages) could be generalized to predict the cluster memberships of future outbreaks, enabling the construction of an improved typing scheme for *Salmonella* outbreak cluster detection. Hence, in addition to evaluating model performance on unseen data by cross-fold validation—the rotation of subsets of the training data as test sets, we further examined the predictive performance of MGEs inferred to be an important class of features by our models in a benchmark against existing gold standard typing schema. The benchmark is based on a set of outbreaks (*N* = 34) completely excluded from model training, that is validation set to evaluate the ability to accurately segregate unseen outbreak sequences by clustering MGE-derived unitigs.

### Study data set

To construct a ground truth data set for model training, we searched for *Salmonella* outbreaks described in the literature and genomic surveillance databases. Literature curation involved identifying peer-reviewed publications in PubMed Central that conducted retrospective epidemiological investigations of *Salmonella* outbreaks by WGS analysis. Qualified publications must provide open access to the WGS data of the isolates and metadata tables describing case cluster information (for example, see references 36, 71–75). Most outbreak cluster labels referenced herein were retained exactly as how they were reported in the original articles. The availability of sampling information, such as geographical location, isolation source, and collection dates, was considered optional.

Compiling the sample metadata necessary for model training and validation was non-trivial. The challenge and complexity stemmed from the heterogeneous representation of sample metadata in journal publications that could take on the form of structured (e.g., Excel sheets, JSON, tables embedded within text) or unstructured (e.g., Images, text) data. Moreover, the inconsistent naming of metadata fields across studies required careful interpretation of individual fields to combine data sets from multiple sources into a single, unified data set. The outcome of an inadequately standardized data ecosystem led to our inefficient use of research resources to support the painstaking process of manually extracting and interpreting the published results and encoding the data in a machine-readable format.

Additional *Salmonella* outbreaks were curated from NCBI Pathogen Detection (25), the process of which was significantly more streamlined. The bacterial genomic surveillance system recently introduced an “outbreak” metadata field to allow sequence submitters to report outbreak cluster information associated with biosamples as free-text values. We treated each unique value in the outbreak field as an independent outbreak cluster. The full Pathogen Detection isolate metadata table was downloaded via the NCBI FTP server (https://ftp.ncbi.nlm.nih.gov/pathogen/Results/Salmonella/PDG000000002.2360/Metadata/) on 30 September 2021.

To control for the minimum discriminatory power required to resolve the outbreak clusters, the study data set included a minimum of two independent outbreak clusters for each serovar predicted by SISTR (76). The final outbreak data set included 343 *Salmonella* isolates associated with 58 outbreaks, subdivided into training and validation sets based on outbreak sample size (*N*). Outbreaks with *N* ≥ 5 were assigned to the training set, tallying up to 239 genomes sequenced from 24 outbreaks associated with six serovars. Outbreaks with 2 ≤ *N* ≤ 4 were assigned to the validation set, tallying up to 104 genomes sequenced from 34 outbreaks associated with six serovars, four of which were unseen in the training set.

All of the genomes analyzed in this study are publicly accessible from NCBI GenBank. The sequence metadata and FTP links to the genome assemblies and annotation files are compiled in Table SA2. The training and validation data sets were retrieved from GenBank on 19 November 2021 and 10 May 2022, respectively. The complete genomic data set including the metadata is also archived in our institutional research data repository: https://doi.org/10.20383/103.0884.

### Building compacted de Bruijn graph

To generate an input variant matrix for machine learning, de Bruijn graphs (dBGs) were constructed from the training and validation draft genome assemblies using Bifrost (27), which connects overlapping *k*-mers, subsequences of a fixed length *k*, to build a directed graph composed of nodes (*k*-mers) and edges. Bifrost stores the source of each *k*-mer as a node attribute called “colors,” which can be extracted from dBGs to quantify the frequency of any subsequence in a population of genomes (27). To minimize graph complexity and the redundancy of the information encoded in the graph, Bifrost compacts dBGs by collapsing non-branching linear paths, forming unitigs and cdBGs. Variant matrices, *M_np_*, encoding presence (*M_np_* = 1) or absence (*M_np_* = 0) of the *p*th unitig in the *n*-th genome, were generated for the training and validation sets using the Bifrost *query* subcommand with the option -e 1. In our clustering benchmark, cdBGs were separately constructed for each serovar in the training and validation data set, and only genomes with N50 score >100 kbps were included to mitigate the effects of assembly contiguity on unitig profile difference.

### Elastic net multinomial logistic model training and evaluation

To quantify the association between genetic variants and outbreak classes, we selected generalized linear model as our model of choice due to the interpretability of coefficient estimates, relatively fast training and prediction time (77–79) and its broad application in many diverse GWAS (68, 80–83). More specifically, we employed multivariable logistic regression to simultaneously estimate the effect size of all features. While the joint analysis of multiple variants generally yields more accurate coefficient estimates due to the ability to model complex interactions or combined effects between multiple variables, care must be taken to account for possible correlation structures among features (70). Given a 2D feature matrix *X_ij_* with a *n*-length vector of outbreak cluster labels (the response) containing *K* classes, we defined a multinomial logistic model as follows:


$$Y_{k}=\beta_{0k}+\sum_{i\;=\;1}^{p}\beta_{ik}X_{i}$$

The dependent variable, *Y_k_*, is the log odd ratio of cluster *k,* where k=1,2,...,K−1. β*_k_* is the vector of regression coefficients with length *p*. To account for the correlation structure between strains of the same lineage, the first two principal components estimated from the cgMLST distance matrix of the training genomes were included as covariates in the model.

To select the most influential features of the response and constrain the effect of noise on model fit, we used the glmnet R package to regularize logistic regression models. For each alpha value, we performed CV by dividing the training data into 60 subsets that were iteratively held out as test data to evaluate model generalizability at different alpha from 0 to 1.0 at steps of 0.1. Model performance (e.g., precision, recall, etc.) at each alpha was evaluated using the caret R package (84). To equally weigh the performance of each class, we reported macro-averaged performance measures at each alpha, calculated as the arithmetic mean of the performance on the outbreak classes.

We calculated RFS to estimate the importance of individual features by computing the fraction of CV folds that each feature was assigned a nonzero coefficient estimated at different alpha levels. The macro-averaged *F*1 score weighted the feature retention frequency at a given alpha level to allocate greater importance to features retained by good performance models. Given regularized models trained at *q* different alpha levels, each associated with a macro-averaged performance score, *w_q_,* and a feature *f* associated with a binary vector, *x_qf_* with length *k* encoding whether *f* was assigned a nonzero coefficient in the *k*th fold at the *q*th alpha level, RFS can be computed as the product of the *x_qf_* vector sum and *w_q_*, summed across *q* and normalized by the total CV folds.


$${RFS}_{f}\;=\;\frac{1}{qk}\sum_{i\;=\;1}^{q}w_{i}\sum_{j\;=\;1}^{k}x_{ijf}$$

## SUPPLEMENTAL MATERIAL

File S1 mbio.02650-24-s0001.docx Additional materials and methods.

File S2 mbio.02650-24-s0002.txt DNA sequences of model-selected unitigs.

Supplemental Figures mbio.02650-24-s0003.pdf Figures SA1 to SA8.

Captions mbio.02650-24-s0004.txt Captions for supplemental materials excluding supplemental figures.

Table SA1 mbio.02650-24-s0005.csv Descriptive summary of the study outbreaks.

Table SA2 mbio.02650-24-s0006.csv Detailed contextual data of the outbreak genomes analyzed in the study.

Table SA3 mbio.02650-24-s0007.csv Functional annotations and genomic origins of the model-selected unitigs.

ASM does not own the copyrights to Supplemental Material that may be linked to, or accessed through, an article. The authors have granted ASM a non-exclusive, world-wide license to publish the Supplemental Material files. Please contact the corresponding author directly for reuse.
