# Supplementary material for: Machine learning reveals the dynamic importance of accessory sequences for Salmonella outbreak clustering
Source: mBio. 2025 Jan 28;16(3):e02650-24. doi: 10.1128/mbio.02650-24 (PMC11898705; doi:10.1128/mbio.02650-24)
Supplement: File S1 — Additional materials and methods. [file mbio.02650-24-s0001.docx]

**Supplemental Materials File 1: Additional Materials and Methods**

## **1.1 Mobilome and unitig annotation**

Plasmid contigs were annotated and clustered using MOB-suite [(1)](https://paperpile.com/c/Y8BPqK/9hgf5). The cluster identifiers assigned to each plasmid by MOB-suite were treated as independent taxonomic units to infer the presence and absence of plasmids in bacterial genomes.

Phage contigs were annotated using Virsorter2 [(2)](https://paperpile.com/c/Y8BPqK/8Ij2z) with a minimum phage sequence length of 5 Kbps. Near-complete viral contigs classified as ‘full’ by Virsorter2 that do not carry any bacterial sequences at the ends of the contigs were annotated as free phages. All remaining viral sequences were classified as prophages. Host sequences found at the boundaries of prophage sequences were trimmed using CheckV [(3)](https://paperpile.com/c/Y8BPqK/ZruTP). To infer orthology between the phage sequences, a sequence similarity matrix was first computed using Dashing [(4)](https://paperpile.com/c/Y8BPqK/FzNZW), then transformed using t-SNE [(5)](https://paperpile.com/c/Y8BPqK/SmTNQ) and finally clustered by HDBSCAN [(6)](https://paperpile.com/c/Y8BPqK/L7zFc).

The *Salmonella* CRISPR locus 1 and 2 were identified using thermonucleotideBLAST [(7)](https://paperpile.com/c/Y8BPqK/WF8wD). The tool performs local alignment similarity search (*in silico* PCR) on genome assemblies using primer pairs designed to amplify CRISPR loci as query sequences [(8)](https://paperpile.com/c/Y8BPqK/L07JL):

CRISPR-1F: 5’-GTRGTRCGGATAATGCTGCC-3’

CRISPR-1R: 5’-CGTATTCCGGTAGATBTDGATGG-3’

CRISPR-2F: 5’-GAGCAATACYYTRATCGTTAACGCC-3’

CRISPR-2R: 5’-GTTGCDATAKGTYGRTRGRATGTRG-3’

The CRISPR ‘amplicons’ identified by *in silico* PCR were supplied as input to CRISPRCasTyper [(9)](https://paperpile.com/c/Y8BPqK/coyN4) to locate spacer and repeat sequences in CRISPR arrays.

Genomic islands (GIs) were annotated from draft genome assemblies and clustered using IslandCompare [(10)](https://paperpile.com/c/Y8BPqK/bkKwS). Polymorphisms within each GI cluster were detected by variant calling using Snippy (<https://github.com/tseemann/snippy>) with reference sequences arbitrarily selected from each GI cluster.

Unitig sequences were extracted from compacted de Bruijn graphs in FASTA format using gfatools (<https://github.com/lh3/gfatools>). Unitig annotation involved finding exact matches of the unitig sequences in input assemblies using bwa fastmap [(11)](https://paperpile.com/c/Y8BPqK/PxYDO). The function of the sequences mapped by unitigs was inferred by intersecting the unitig alignment coordinates in BED format with the annotated feature coordinates in GFF format using BEDTools [(12)](https://paperpile.com/c/Y8BPqK/Ii89M). A minimum overlap of 10 bps between the unitig alignment and feature interval was required to qualify as a valid intersection.

## **1.2 Phylogenomic analysis**

cgMLST and wgMLST profiles were generated using chewieSnake [(13)](https://paperpile.com/c/Y8BPqK/f9z2j), an end-to-end MLST analysis workflow that wraps around chewBBACA [(14)](https://paperpile.com/c/Y8BPqK/3cwJc) to perform gene prediction and allele calling based on user-provided MLST schema. The *Salmonella* cgMLST (3,000 loci) and wgMLST (8,558 loci) schema were downloaded from <https://zenodo.org/record/4724927> and <https://zenodo.org/record/1323684>, respectively. Pairwise hamming distances were calculated using a custom R script and clustered by neighbour-joining (NJ) in R using the ape R package [(15)](https://paperpile.com/c/Y8BPqK/JHsqt). NJ trees were rooted using the phangorn R package [(16)](https://paperpile.com/c/Y8BPqK/IPIz9). MonoPhylo [(17)](https://paperpile.com/c/Y8BPqK/pjtkO) was used to test if a vector of tree tips formed monophyletic groups in rooted trees, thereby enabling the calculation of monophyletic rates. Core genome single nucleotide variant (cgSNV) alignment of the training genomes was generated by mapping the assembly contigs to the ser. Typhimurium LT2 reference sequence using Snippy (<https://github.com/tseemann/snippy>). Recombinant and uninformative (monomorphic) sites were filtered from the cgSNV alignment using Gubbins [(18)](https://paperpile.com/c/Y8BPqK/ihfJ6) and snp-sites [(19)](https://paperpile.com/c/Y8BPqK/YvuRH), respectively. The filtered cgSNV alignment was provided as input to RAxML-NG [(20)](https://paperpile.com/c/Y8BPqK/k8hG3) to construct a maximum likelihood tree with 1,000 bootstrap replicates under the assumption of the general time-reversible nucleotide model with gamma distribution for rate heterogeneity. Maximum likelihood and NJ trees were visualized in R using the ggtree R package [(21)](https://paperpile.com/c/Y8BPqK/NDgTh).

# **References**

1. [Robertson J, Nash JHE. MOB-suite: software tools for clustering, reconstruction and typing of plasmids from draft assemblies. Microb Genom [Internet]. 2018 Aug;4(8). Available from:](http://paperpile.com/b/Y8BPqK/9hgf5) <http://dx.doi.org/10.1099/mgen.0.000206>

2. [Guo J, Bolduc B, Zayed AA, Varsani A, Dominguez-Huerta G, Delmont TO, et al. VirSorter2: a multi-classifier, expert-guided approach to detect diverse DNA and RNA viruses. Microbiome. 2021 Feb 1;9(1):37.](http://paperpile.com/b/Y8BPqK/8Ij2z)

3. [Nayfach S, Camargo AP, Schulz F, Eloe-Fadrosh E, Roux S, Kyrpides NC. CheckV assesses the quality and completeness of metagenome-assembled viral genomes. Nat Biotechnol. 2021 May;39(5):578–85.](http://paperpile.com/b/Y8BPqK/ZruTP)

4. [Baker DN, Langmead B. Dashing: fast and accurate genomic distances with HyperLogLog. Genome Biol. 2019 Dec 4;20(1):265.](http://paperpile.com/b/Y8BPqK/FzNZW)

5. [van der Maaten L, Hinton G. Visualizing Data using t-SNE. J Mach Learn Res. 2008;9(86):2579–605.](http://paperpile.com/b/Y8BPqK/SmTNQ)

6. [McInnes L, Healy J, Astels S. hdbscan: Hierarchical density based clustering. J Open Source Softw. 2017 Mar 21;2(11):205.](http://paperpile.com/b/Y8BPqK/L7zFc)

7. [Gans JD, Wolinsky M. Improved assay-dependent searching of nucleic acid sequence databases. Nucleic Acids Res. 2008 Jul;36(12):e74.](http://paperpile.com/b/Y8BPqK/WF8wD)

8. [Yang L, Zhang X, Liu Y, Li H, Qiu S, Li P, et al. CSESA: an R package to predict Salmonella enterica serotype based on newly incorporated spacer pairs of CRISPR. BMC Bioinformatics. 2019 Apr 27;20(1):215.](http://paperpile.com/b/Y8BPqK/L07JL)

9. [Russel J, Pinilla-Redondo R, Mayo-Muñoz D, Shah SA, Sørensen SJ. CRISPRCasTyper: Automated Identification, Annotation, and Classification of CRISPR-Cas Loci. CRISPR J. 2020 Dec;3(6):462–9.](http://paperpile.com/b/Y8BPqK/coyN4)

10. [Bertelli C, Gray KL, Woods N, Lim AC, Tilley KE, Winsor GL, et al. Enabling genomic island prediction and comparison in multiple genomes to investigate bacterial evolution and outbreaks. Microb Genom [Internet]. 2022 May;8(5). Available from:](http://paperpile.com/b/Y8BPqK/bkKwS) <http://dx.doi.org/10.1099/mgen.0.000818>

11. [Li H, Durbin R. Fast and accurate long-read alignment with Burrows-Wheeler transform. Bioinformatics. 2010 Mar 1;26(5):589–95.](http://paperpile.com/b/Y8BPqK/PxYDO)

12. [Quinlan AR. BEDTools: The Swiss-Army Tool for Genome Feature Analysis. Curr Protoc Bioinformatics. 2014 Sep 8;47:11.12.1–34.](http://paperpile.com/b/Y8BPqK/Ii89M)

13. [Deneke C, Uelze L, Brendebach H, Tausch SH, Malorny B. Decentralized Investigation of Bacterial Outbreaks Based on Hashed cgMLST. Front Microbiol. 2021 May 28;12:649517.](http://paperpile.com/b/Y8BPqK/f9z2j)

14. [Silva M, Machado MP, Silva DN, Rossi M, Moran-Gilad J, Santos S, et al. chewBBACA: A complete suite for gene-by-gene schema creation and strain identification. Microb Genom [Internet]. 2018 Mar;4(3). Available from:](http://paperpile.com/b/Y8BPqK/3cwJc) <http://dx.doi.org/10.1099/mgen.0.000166>

15. [Paradis E, Claude J, Strimmer K. APE: Analyses of Phylogenetics and Evolution in R language. Bioinformatics. 2004 Jan 22;20(2):289–90.](http://paperpile.com/b/Y8BPqK/JHsqt)

16. [Schliep KP. phangorn: phylogenetic analysis in R. Bioinformatics. 2011 Feb 15;27(4):592–3.](http://paperpile.com/b/Y8BPqK/IPIz9)

17. [Portik DM, Wiens JJ. Do Alignment and Trimming Methods Matter for Phylogenomic (UCE) Analyses? Syst Biol. 2021 Apr 15;70(3):440–62.](http://paperpile.com/b/Y8BPqK/pjtkO)

18. [Croucher NJ, Page AJ, Connor TR, Delaney AJ, Keane JA, Bentley SD, et al. Rapid phylogenetic analysis of large samples of recombinant bacterial whole genome sequences using Gubbins. Nucleic Acids Res. 2015 Feb 18;43(3):e15.](http://paperpile.com/b/Y8BPqK/ihfJ6)

19. [Page AJ, Taylor B, Delaney AJ, Soares J, Seemann T, Keane JA, et al. SNP-sites: rapid efficient extraction of SNPs from multi-FASTA alignments. Microb Genom. 2016 Apr;2(4):e000056.](http://paperpile.com/b/Y8BPqK/YvuRH)

20. [Hübner L, Kozlov AM, Hespe D, Sanders P, Stamatakis A. Exploring parallel MPI fault tolerance mechanisms for phylogenetic inference with RAxML-NG. Bioinformatics. 2021 Nov 18;37(22):4056–63.](http://paperpile.com/b/Y8BPqK/k8hG3)

21. [Yu G, Lam TTY, Zhu H, Guan Y. Two Methods for Mapping and Visualizing Associated Data on Phylogeny Using Ggtree. Mol Biol Evol. 2018 Dec 1;35(12):3041–3.](http://paperpile.com/b/Y8BPqK/NDgTh)
