# Supplementary material for: Machine learning reveals the dynamic importance of accessory sequences for Salmonella outbreak clustering
Source: mBio. 2025 Jan 28;16(3):e02650-24. doi: 10.1128/mbio.02650-24 (PMC11898705; doi:10.1128/mbio.02650-24)
Supplement: Supplemental Figures — Figures SA1 to SA8. [file mbio.02650-24-s0003.pdf]

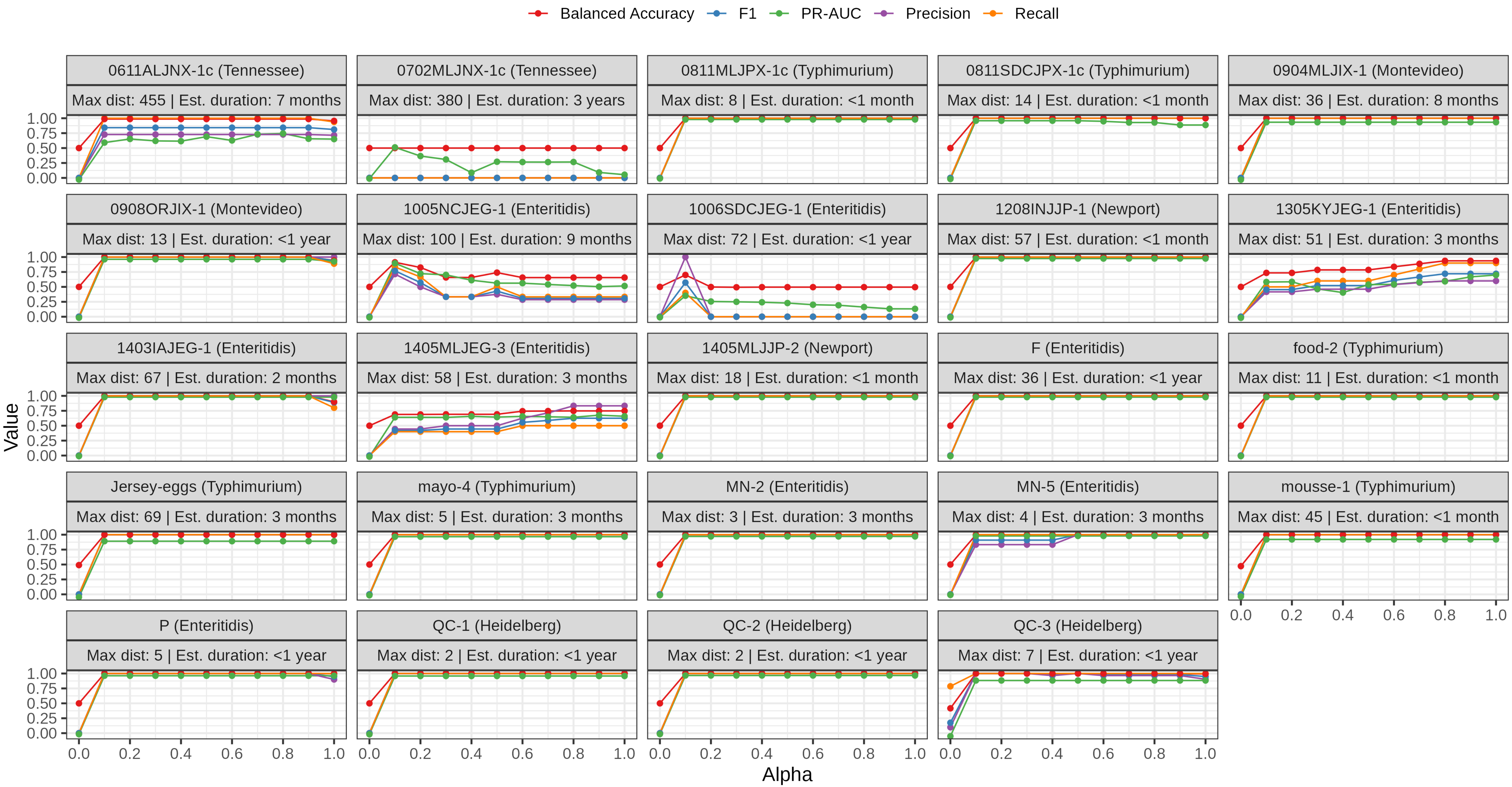

Figure A1. Model performance on individual outbreak classes covaried with outbreak clonality and outbreak duration. Model prediction error rate increased with maximum pairwise distance (D) and outbreak duration. Superior model performance was observed in short-lived (estimated duration ≤1 month) and highly clonal (D < 50) outbreaks. Outbreak duration was inferred from sample collection date ranges reported at month or day level. Outbreaks with unreported collection dates or dates reported at the year level were designated to have unknown durations.

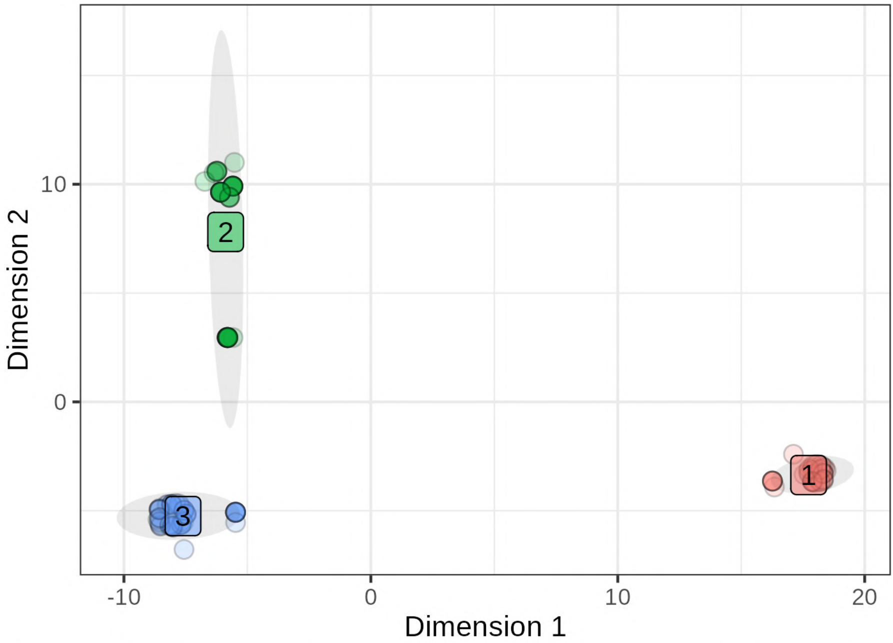

Figure A2. Identification of three phage orthologous groups by t-SNE analysis in three point-source foodborne outbreaks caused by Salmonella ser. Heidelberg in the Province of Quebec, Canada. t-SNE was performed on the pairwise Jaccard distance matrix of phage sequences identified by Virsorter2. The reduced dimensions of the phage sequence similarity matrix were clustered into orthologous groups using HDBSCAN.

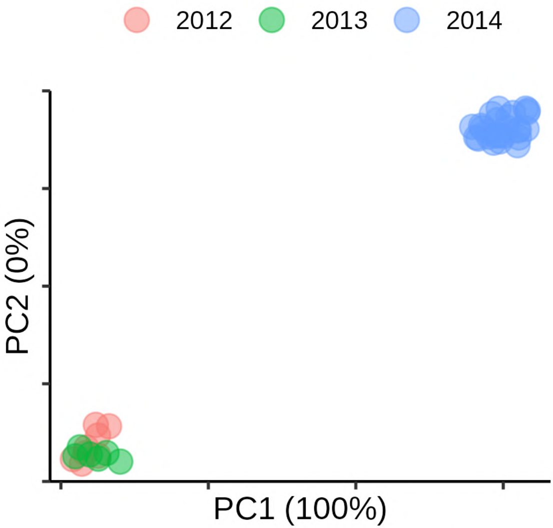

Figure A3. Identification of a recombinant allele in a Salmonella pathogenicity island (SPI) found conserved across all isolates from the three point-source foodborne outbreaks in the Province of Quebec, Canada. Hundreds of polymorphic sites were found to span over a 2 Kbps region in the SPI, forming two distinct haplotypes, one of which is unique to the 2014 outbreak, as shown by the principal component analysis on the single nucleotide variant (SNV) matrix of the SPI. The axes represent the first two principal components; however, the first principal component was sufficient to explain 100% of the variance in the SNV matrix.

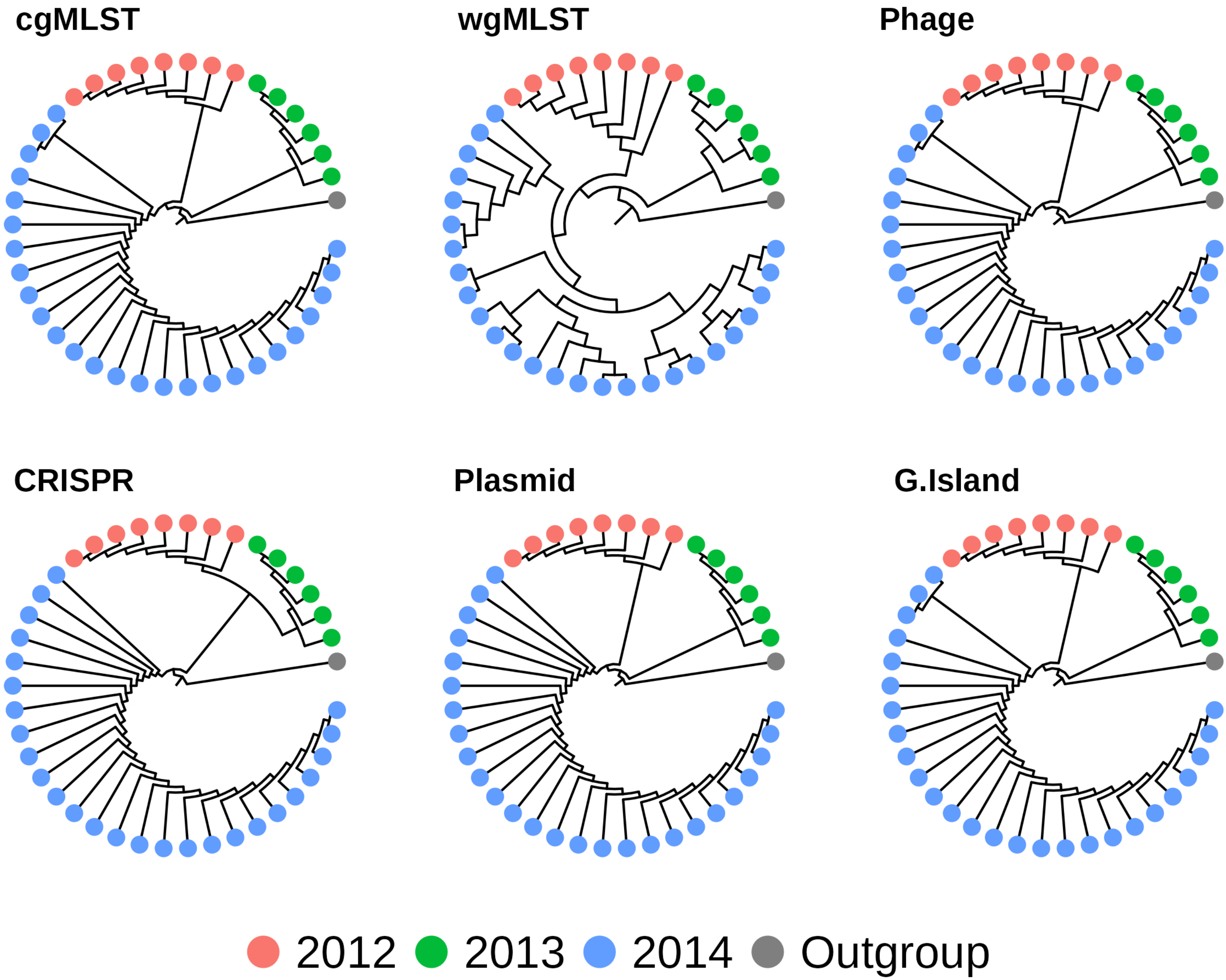

Figure A4. Topological comparison of rooted neighbour-joining trees constructed by existing systematics methods (cgMLST and wgMLST) and mobile genetic elements (MGEs) analysis. In all instances, the three Heidelberg outbreaks occurring between 2012-2014 formed monophyletic clades, suggesting the feasibility of outbreak discrimination by MGE typing. The neighbour-joining trees were generated using the ape R package and visualized using the ggtree R package.

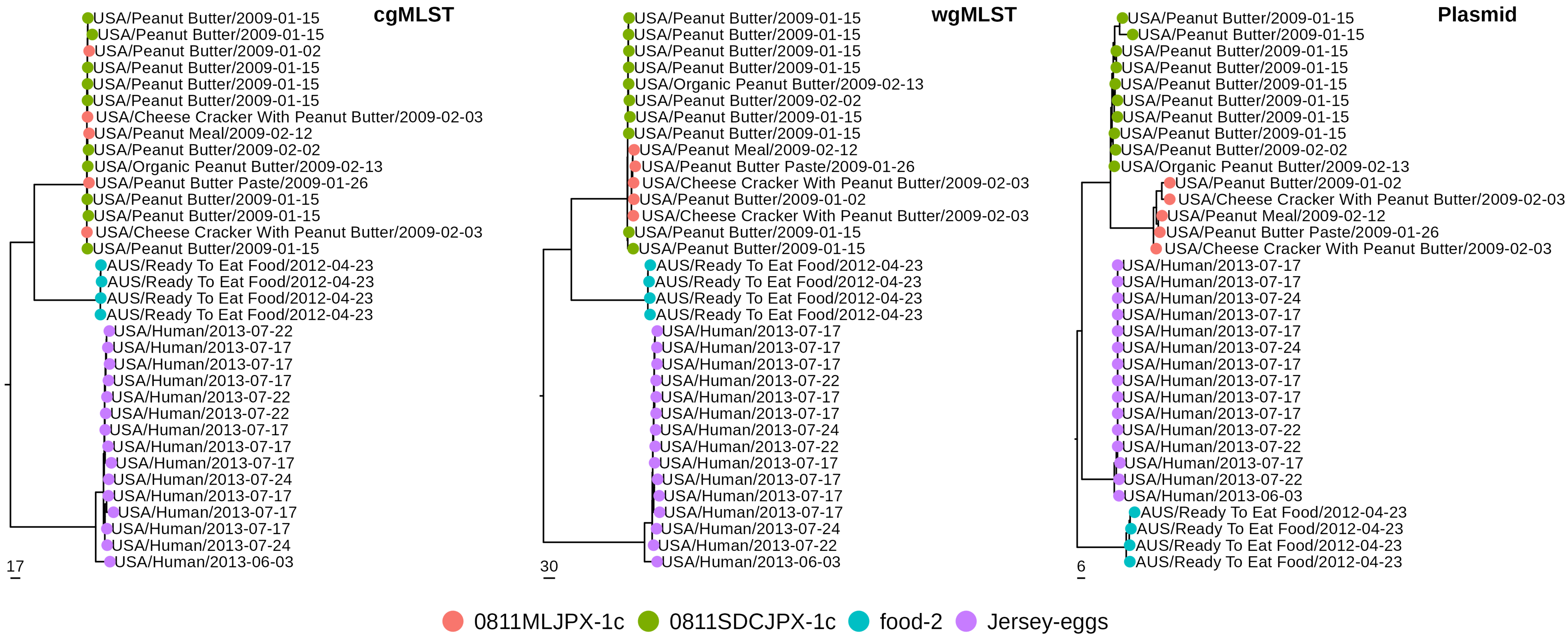

Figure A5. The inclusion of accessory sequence variations resulted in superior discriminatory power. Core genome differences were insufficient to discriminate two ser. Typhimurium outbreaks, labelled "0811MLJPX-1c" and "0811SDCJPX-1c". Genotyping the isolates at the whole-genome level led to the segregation of the two outbreaks into distinct clades. Measurable differences were found in plasmid sequences, which correlated with the elevated resolution in wgMLST. The cgMLST and wgMLST alleles were called based on two MLST schema consisting of 3,000 and 8,558 loci, respectively. Neighbour-joining (NJ) trees were constructed from the allele distance matrices using the ape R package. The plasmid dendrogram was constructed by building a compacted de Bruijn graph from the Typhimurium genomes in the training dataset and identifying unitigs that mapped to plasmid contigs. The binary profiles of the selected unitigs were clustered by the NJ algorithm to construct the plasmid dendrogram. The trees were collectively rooted by an outgroup (GenBank Accession: GCA_010689465.1) and visualized using the ggtree R package.

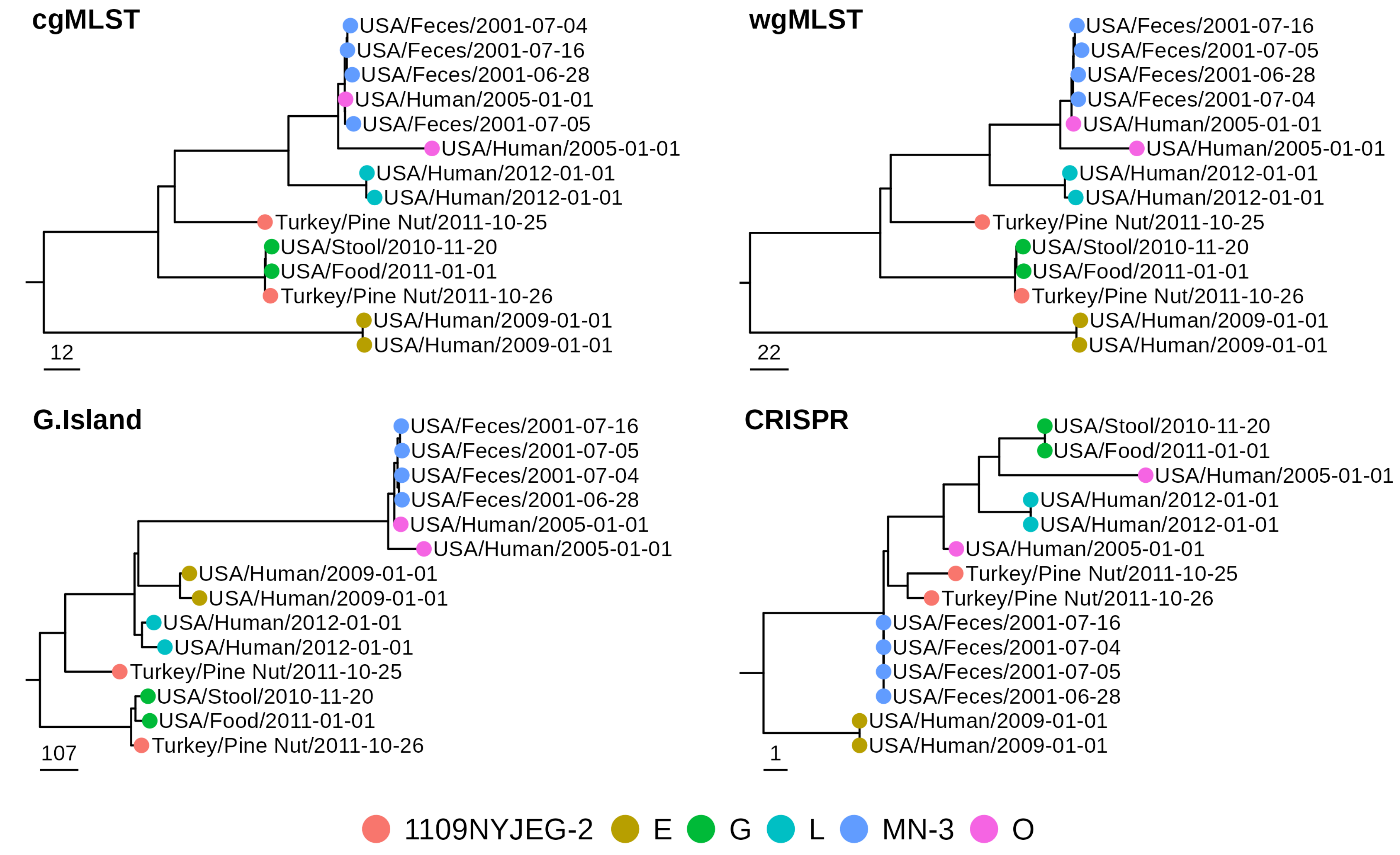

Figure A6. Two scenarios in which MGE typing led to improved outbreak clustering performance. In the first scenario, core genome differences were insufficient to discriminate between outbreak “MN-3” isolates and an isolate from outbreak “O”. Genotyping the isolates by accessory sequence variations or at the whole genome level (wgMLST) resulted in the monophyletic clustering of the MN-3 isolates. In the second scenario, cgMLST and wgMLST identified two nut isolates from outbreak “1109NYJEG-2” as distant relatives, forming a polyphyletic group. When clustering the two nut isolates by CRISPR unitigs, the isolates formed a monophyletic group in the CRISPR dendrogram, suggesting that the two isolates shared conserved molecular signatures in their CRISPR arrays. The cgMLST and wgMLST alleles were called based on two MLST schema consisting of 3,000 and 8,558 loci, respectively. Neighbour-joining (NJ) trees were constructed from the allele distance matrices using the ape R package. The CRISPR and genomic island (GI) dendrograms were constructed by building a compacted de Bruijn graph from the Enteritidis genomes in the validation dataset and identifying unitigs that mapped to CRISPR or GI contigs. The binary profiles of the selected unitigs were clustered by the NJ algorithm to construct CRISPR and GI dendrograms. The trees were collectively rooted by an outgroup (GenBank Accession: GCA_008584365.1) and visualized using the ggtree R package.

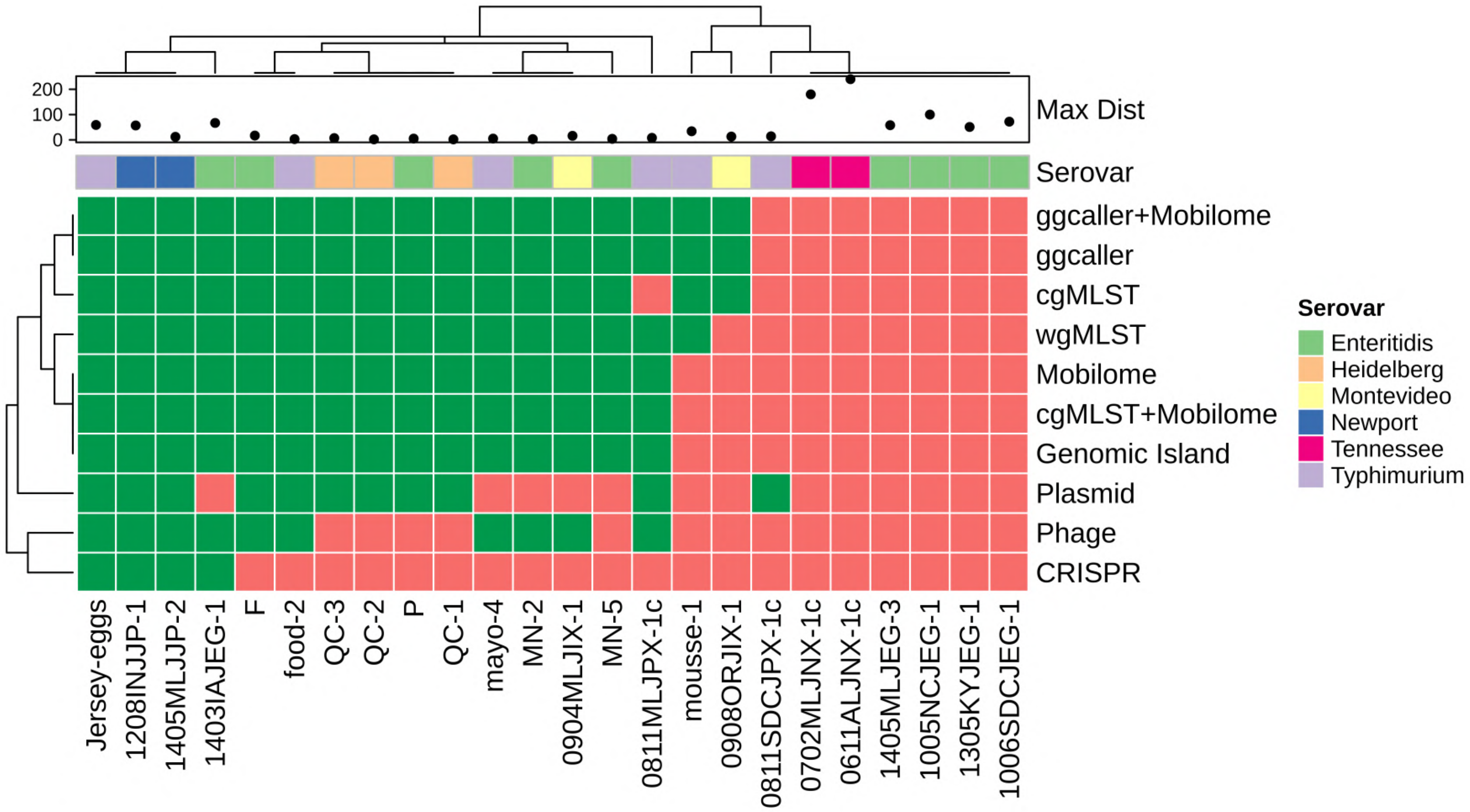

Figure A7. The effect of the maximum pairwise distance of outbreaks on the monophyletic clustering of training outbreak clusters (N = 24) in dendrograms constructed by different methods. The formation of monophyletic clades (green) is inversely correlated with the maximum pairwise distance of an outbreak (measured in cgMLST distance). Outbreaks with larger linkage distances frequently failed to form monophyletic clades, irrespective of the methods used to construct the dendrogram. Monophyletic, paraphyletic, and polyphyletic clustering of a vector of tips in a given tree was assessed using MonoPhylo. The outbreak cluster labels were retained exactly as how they were reported in the original sources.

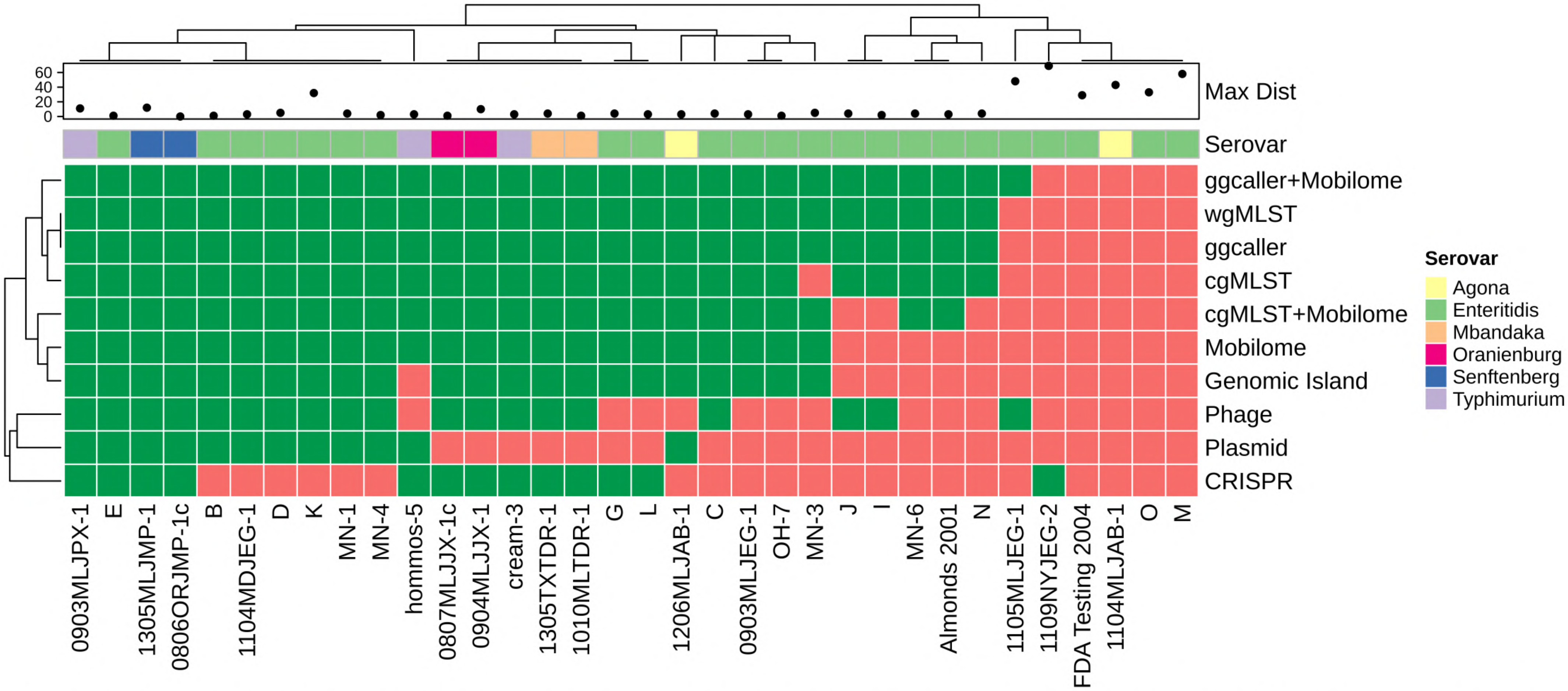

Figure A8. The effect of the maximum pairwise distance of outbreaks on the monophyletic clustering of validation outbreak clusters (N = 34) in dendrograms constructed by different methods. The formation of monophyletic clades (green) is inversely correlated with the maximum pairwise distance of an outbreak (measured in cgMLST distance). Outbreaks with larger linkage distances frequently failed to form monophyletic clades, irrespective of the methods used to construct the dendrogram. Monophyletic, paraphyletic, and polyphyletic clustering of a vector of tips in a given tree was assessed using MonoPhylo. The outbreak cluster labels were retained exactly as how they were reported in the original sources.
